# Comparison of sequencing data processing pipelines and application to underrepresented African human populations

**DOI:** 10.1186/s12859-021-04407-x

**Published:** 2021-10-09

**Authors:** Gwenna Breton, Anna C. V. Johansson, Per Sjödin, Carina M. Schlebusch, Mattias Jakobsson

**Affiliations:** 1 Human Evolution, Department of Organismal Biology, Evolutionary Biology Centre, Uppsala University, Norbyvägen 18C, 752 36 Uppsala, Sweden; 2 Department of Cell and Molecular Biology, National Bioinformatics Infrastructure Sweden, Science for Life Laboratory, Uppsala University, Husargatan 3, 752 37 Uppsala, Sweden; 3 Palaeo-Research Institute, University of Johannesburg, P.O. Box 524, Auckland Park, 2006 South Africa; 4 Science for Life Laboratory, Uppsala, Sweden

**Keywords:** Genome Analysis Toolkit (GATK), High-throughput sequencing (HTS), Next generation sequencing (NGS), High coverage genomes, Underrepresented ancestry, Comparison of pipelines

## Abstract

**Background:**

Population genetic studies of humans make increasing use of high-throughput sequencing in order to capture diversity in an unbiased way. There is an abundance of sequencing technologies, bioinformatic tools and the available genomes are increasing in number. Studies have evaluated and compared some of these technologies and tools, such as the Genome Analysis Toolkit (GATK) and its “Best Practices” bioinformatic pipelines. However, studies often focus on a few genomes of Eurasian origin in order to detect technical issues. We instead surveyed the use of the GATK tools and established a pipeline for processing high coverage full genomes from a diverse set of populations, including Sub-Saharan African groups, in order to reveal challenges from human diversity and stratification.

**Results:**

We surveyed 29 studies using high-throughput sequencing data, and compared their strategies for data pre-processing and variant calling. We found that processing of data is very variable across studies and that the GATK “Best Practices” are seldom followed strictly. We then compared three versions of a GATK pipeline, differing in the inclusion of an indel realignment step and with a modification of the base quality score recalibration step. We applied the pipelines on a diverse set of 28 individuals. We compared the pipelines in terms of count of called variants and overlap of the callsets. We found that the pipelines resulted in similar callsets, in particular after callset filtering. We also ran one of the pipelines on a larger dataset of 179 individuals. We noted that including more individuals at the joint genotyping step resulted in different counts of variants. At the individual level, we observed that the average genome coverage was correlated to the number of variants called.

**Conclusions:**

We conclude that applying the GATK “Best Practices” pipeline, including their recommended reference datasets, to underrepresented populations does not lead to a decrease in the number of called variants compared to alternative pipelines. We recommend to aim for coverage of > 30X if identifying most variants is important, and to work with large sample sizes at the variant calling stage, also for underrepresented individuals and populations.

**Supplementary Information:**

The online version contains supplementary material available at 10.1186/s12859-021-04407-x.

## Background

Describing and understanding diversity has been a focus of biology for a long time. In particular, genetic diversity is informative about the demographic and selective processes which have shaped all species—including humans. Over the last few decades, our understanding of genetic diversity has increased dramatically, thanks to methodological developments. However, many methods—such as single nucleotide polymorphisms (SNPs) arrays—suffer from ascertainment bias and bias towards known variants. This is particularly problematic when investigating diversity in non-model organisms or in populations highly diverged from the population(s) the SNPs were discovered in. In humans, ascertainment bias is limiting our understanding of the diversity in populations of Sub-Saharan African ancestry. The development of resequencing and high-throughput sequencing (HTS) technologies enabled us to come closer to the “true” diversity while being more affordable and less time intensive than other methods such as Sanger sequencing of entire genomes or de novo assemblies. However, even if we assume that all the necessary information about an individual’s genome is contained in the raw HTS data (i.e. in the raw reads), there are many steps and decisions between that raw data and a set of non-ascertained variants. The key steps are: mapping to a reference genome; quality control and processing of the resulting files (often in BAM format); variant calling; and callset refinement. Numerous softwares and algorithms are available to perform each of these tasks [1, 2].

The challenge can be daunting, both when deciding on a workflow to process new HTS data, and when assembling a comparative dataset to put the new results into perspective. There are two main options in terms of assembling a comparative dataset: either the data is provided as the end result (i.e. a variant file, often in VCF format) or the raw data is available. In the latter case, this means that the data has to be processed again, a computationally intensive and time-consuming process. At the same time, we are starting to realize that combining datasets at the end stage (VCF) without accounting for differences in the processing workflows can lead to biases and signals which have no biological meaning but are solely due to differences in sequencing technologies or processing steps [3].

There is presently a deficiency of comparisons of processing workflows, and thus many questions remain open in terms of which choices matter. A few studies have compared and evaluated workflows, e.g. Hwang et al*.* [4] compared seven short-read mappers and ten variant callers (including three Genome Analyses Toolkit (GATK) [5] variant callers) on whole genome data for two individuals. The authors focus on minimizing false negatives and work with only two individuals (one of European and one of Sub-Saharan African ancestry). One of the results was that the common combination of alignment with bwa [6] (mem algorithm) and variant calling with GATK’s HaplotypeCaller (HC) does not perform worse compared to other methods—for example methods combining several variant callers. Another study [7] focused on establishing a standard BAM processing pipeline. However, the focus in this study was less on the development of the pipeline than on evaluating whether the pipeline run at different sequencing centers gives the same results. Moreover, it was targeted at very large datasets (tens of thousands of genomes), a sample size that few studies obtain. In a third study [8], different workflows were applied to a dataset of low-coverage genomes; the union of the callsets is the input for downstream filtering and analyses (together with data from high coverage genomes, exomes and SNP arrays).

In this study, we focus on the effect of applying different workflows on a dataset consisting of 28 high-coverage genomes (minimum depth after processing: 18.9X). Moreover, we chose to focus on the GATK, a set of tools to discover variants in HTS data. The GATK provides tools to perform different tasks, and proposes “Best Practices workflows” that are developed specifically for certain types of data, such as the “Germline short variant discovery (SNPs + Indels)” [9, 10]. In the following, when writing the “Best Practices workflow” we refer to the “Germline short variant discovery (SNPs + Indels)” workflow. The Best Practices workflow details the different steps of processing pipelines, with the associated tools (of which not all are GATK tools) and parameter values. One advantage of following the GATK’s Best Practices workflow is that it is well documented and tested, and used in many studies, in particular those focusing on humans. The Best Practices workflow might however not be optimal for all human studies, as it requires a number of reference datasets that are ascertained towards specific human ancestries, in particular for one step of the BAM processing (Base Quality Score Recalibration, or BQSR) and one callset refinement step (Variant Quality Score Recalibration, or VQSR). Moreover, the Best Practices are constantly evolving, which can complicate the aggregation of data processed using different versions of the Best Practices.

We started by reviewing the processing workflows of 29 HTS studies, most of them using GATK tools. The goal of this review was to investigate whether the Best Practices are followed in practice and which GATK tools are most used. We then compared three HTS processing pipelines on a set of 28 individuals of diverse ancestries—with a focus on Sub-Saharan African populations. We compared the 2019 version of the Best Practices (using GATK version 3), the 2015 version of the Best Practices, and a pipeline that contains most of the Best Practices steps but in which the BQSR step is replaced by a custom BQSR step, the purpose of which was to diminish the possible effect of using ascertained reference datasets. We then compared the last pipeline with an identical pipeline except that it comprised more individuals at the joint genotyping step [11]. Finally, we tested the correlation between coverage and number of variants, to discuss whether all “high coverage” genomes (≥ 20X) are equivalent (for example in terms of number of called variants).

## Results

### Literature survey

We reviewed the processing pipelines of 29 HTS studies, 23 of which focus on human populations and six on other mammals (listed in Table 1).

**Table 1:** List of studies included in the literature survey

Study	Species	Populations
[12]	Human	Malay
[13]	Human	Khoe-San
[8]	Human	Worldwide
[14]	Human	Dane
[15]	Human	Icelandic
[16]	Human	Japanese
[17]	Human	UK
[18]	Human	Qatari
[19]	Human	Chadian, Greek, Lebanese
[20]	Human	Aboriginal Australian
[21]	Human	Worldwide
[22]	Human	Worldwide
[23]	Human	Swede
[24]	Human	South African
[25]	Human	Peruvian
[26]	Human	Korean
[27]	Human	Nepalese
[28]	Human	US, Finn, Estonian
[29]	Human	Japanese
[30]	Human	Various African
[31]	Human	Various African
[32]	Human	North African, Basque, Iraqi
[33]	Human	Worldwide
[34]	Macaque	–
[35]	Wolf, dog	–
[36]	Dog	–
[37]	Dog	–
[38]	Macaque	–
[39]	Green monkey	–

Studies are ordered first by species (Human / other), then by date, and finally by alphabetical order of first author’s last name

We summarized the information for some processing steps in Table 2 (see Additional file 1 for more details): BAM processing (indel realignment and GATK’s BQSR), variant calling (GATK’s HaplotypeCaller (HC) and GenotypeGVCFs or GATK’s UnifiedGenotyper (UG)), and callset recalibration (GATK’s Variant Quality Score Recalibration (VQSR) or hard filtering).

**Table 2:** Overview of the steps in 29 HTS studies

Study	Indel realignment	BQSR^BP^	HC^BP^	UG	Other variant caller	VQSR^BP^	Hard filtering
[12]	No	No	No	No	Yes	No	Yes
[13]	Yes	No	No	No	Yes	No	Yes
[8]	Yes	Yes	NA	NA	NA	NA	NA
[14]^#^	Yes	Yes	Yes	No	No	Yes	No
[15]	Yes	Yes	Maybe^1^	Maybe^1^	No	No	Yes
[16]	No	No	Yes	No	Yes	No	Yes
[17]	Yes	Yes	No	Yes	Yes	Yes	No
[18]	Yes?^2^	Yes?^2^	Maybe^1^	Maybe^1^	No	No	Yes
[19]	NA	NA	No	No	Yes	NA	NA
[20]	Yes	No	No	No	Yes	No	Yes
[21]	No	No	No	Yes	No	No	No
[22]	No	No	No	No	Yes	No	No
[23]^#^	No	Yes	Yes	No	No	Yes	No
[24]^#^	Yes (other)^2^	Yes	Yes	No	Yes	Yes	Yes
[25]	Yes	Yes	Yes	No	No	No	No
[26]	Yes (NA)^2^	Yes (NA)^2^	No	Yes	No	No	Yes
[27]	Yes	Yes	No	No	Yes	No	Yes
[28] cohort 1	No	Yes (other)^2^	No	No	Yes	No	Yes
[28] cohort 2	No	No	Yes	No	No	Yes	Yes
[29]^#^	Yes	Yes	Yes	No	No	Yes	Yes
[30]	No	No	No	Yes	No	No	No
[31]	Yes	Yes	No	Yes	No	Yes	No
[32]	Yes	Yes	No	Yes	No	Yes	No
[33]	No	No	Yes	No	No	Yes	Yes
[34]*	Yes	Yes	No	Yes	No	No	Yes
[35]*	Yes	Yes	No	Yes	No	No	Yes
[36]*^#^	No	Yes	Yes	No	No	Yes	No
[37]*^#^	No	Yes	Yes	No	No	Yes	No
[38]*	Yes	No	Yes	No	No	No	Yes
[39]*	Yes	Yes	Yes	No	No	No	Yes

*BQSR* Base Quality Score Recalibration, *HC* HaplotypeCaller + GenotypeGVCFs, *VQSR* Variant Quality score Recalibration, *UG* UnifiedGenotyper

^BP^GATK tool in the Best Practices in 2019

^*^Species other than human

^#^Reports using BQSR, HC + GenotypeGVCFs and VQSR

^1^Uncertainty as to which GATK variant caller was used (HC or UG)

^2^Various uncertainties or use of alternative software for indel realignment or BQSR

“BQSR” is a step in the BAM processing pipeline, where base quality scores are recalibrated, to correct for biases due to the sequencing. It requires a set of known variants, for example dbSNP [40].

“Hard filtering” designates a callset filtering strategy where variants are kept or removed depending on user defined thresholds for variants’ annotations of interest. VQSR, on the other hand, is an approach that learns the features of “true” variants and gives a score to the remaining variants. It requires several datasets: a “truth resource” (used here: HapMap 3 and polymorphic sites from the Omni 2.5 M SNP array), a “training resource” (used here: 1000G) and a “known sites resource” (used here: dbSNP). The truth and the training resources are used to train the recalibration model which tries to characterize the relationship between the variants’ annotations and the probability that a variant is a true variant or an artefact. The known sites resource is used to stratify metrics (such as the transition to transversion ratio) between variants found in the known sites resource and new variants. The user then decides on a “tranche threshold”. For example, a tranche threshold of 99.9 means that 99.9% of the variants in the truth set will be included—and all of the variants which have a score as high as these 99.9% will pass the filter. For more background, see [9, 10, 41].

Among the 29 HTS studies, the pipelines are very diverse (Table 2 and Additional file 1). Of the 23 studies on human data, only four have the BQSR, HC + GenotypeGVCFs and VQSR steps; of these, three also ran the indel realignment step with GATK while the fourth did it with another software. Of the six studies on other mammals, two have the BQSR, HC + GenotypeGVCFs and VQSR steps. We observe that the majority of the included studies use GATK for at least one step (20 from 23 human studies, six from six studies with other mammals); however this is possibly an effect of our strategy for selecting studies. 13 of the 23 human studies use GATK for at least two steps. Of the human studies, we could determine with certainty that GATK was used for indel realignment in 11 studies; BQSR in 11 studies; HC for eight studies; VQSR for nine studies; and UG for six studies.

### The majority of variants are identical for different BAM processing (for a given set of individuals)

**Fig. 1:**
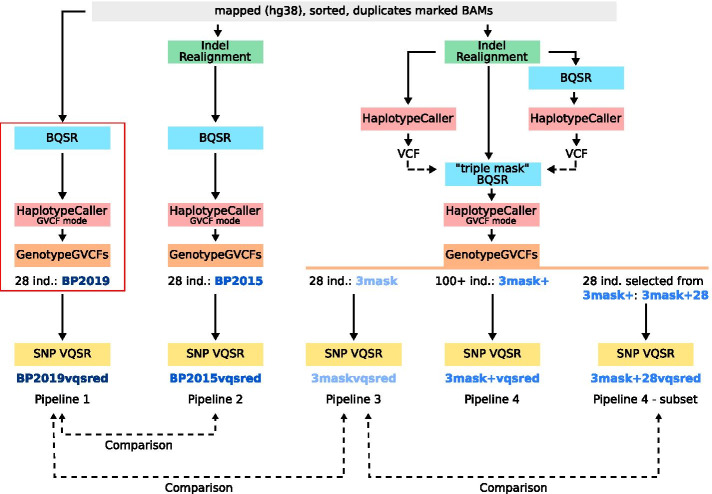
Three (plus one) BAM processing and variants calling pipelines. The dashed lines are relative to the comparisons mentioned in the text. Ind. = individuals

We compared three BAM processing pipelines (pipelines 1, 2 and 3 in Fig. 1). “BP2019” is the output of the Best Practices workflow in 2019. The “BP2015” workflow includes an extra step, indel realignment, corresponding to the Best Practices in 2015. That step is redundant with the HC variant caller (because HC includes local remapping in regions where there seem to be variants) but was not removed from the Best Practices directly after the introduction of HC. The “3mask” workflow has a custom BQSR step (as in [3], and similar to what is done when working with organisms lacking reference datasets [34, 42, 43]). We performed SNP VQSR with a tranche threshold of 99.9 for each of these callsets, resulting in “BP2019vqsred”, “BP2015vqsred” and “3maskvqsred”.

The three pipelines were applied to a set of 28 high coverage genomes (average genome depth, with duplicates, directly after mapping: 19.6X–74.6X, mean across individuals: 39.3X, Additional files 2, 3) [8, 21, 44]. The individuals represent five different ancestries, with a focus on Sub-Saharan African ancestries: the dataset includes six individuals with European background; four Yoruba individuals (western Africa); four Dinka individuals (eastern Africa); seven Khoe-San individuals (five Ju|’hoansi, two #Khomani), representing hunter-gatherers from southern Africa; and seven rainforest hunter-gatherers (two Biaka, five Mbuti) from central Africa.

We collected various metrics of the callsets using Picard's CollectVariantCallingMetrics, before and after VQSR, for the entire callset and for each individual. Some of these metrics are reported in Table 3.

**Table 3:** Metrics in “BP2019”, “BP2015”, and “3mask”, at callset and individual level, before and after VQSR

	Metrics	BP2019	BP2015	3mask
*Counts for the entire callset*				
Before VQSR	Biallelic SNPs	20,301,167	20,301,911	20,312,127
	Multiallelic SNPs	85,510	85,517	85,725
	Simple indels	2,599,873	2,601,041	2,601,657
	Complex indels	737,325	738,010	737,834
	Singletons	7,975,044	7,974,844	7,980,292
	Biallelic SNPs in dbSNP (%)	96.68%	96.68%	96.67%
	Simple indels in dbSNP (%)	94.30%	94.30%	94.29%
After VQSR	Biallelic SNPs	19,619,238	19,596,831	19,591,088
	Multiallelic SNPs	75,300	75,132	75,115
	Singletons (SNPs)	6,930,326	6,921,952	6,923,568
	Filtered SNPs	692,139	715,465	731,649
	Biallelic SNPs in dbSNP (%)	96.87%	96.88%	96.88%
*Average (and standard deviation (stdev)) per individual*				
Before VQSR	Biallelic SNPs (average)	4,443,858.18	4,444,067.04	4,445,566.93
	Biallelic SNPs (stdev)	438,889.45	438,945.07	438,990.07
	Biallelic SNPs (min)	3,442,414	3,442,476	3,443,131
	Biallelic SNPs (max)	4,916,206	4,916,439	4,917,928
	Multiallelic SNPs (average)	32,357.82	32,354.79	32,414.75
	Multiallelic SNPs (stdev)	3943.62	3943.33	3961.22
	Simple indels (average)	508,131.89	508,474.93	508,597.68
	Simple indels (stdev)	46,623.55	46,488.14	46,465.10
	Complex indels (average)	346,817.75	347,534.14	347,547.21
	Complex indels (stdev)	25,452.05	25,499.59	25,401.04
	Singletons (average)	284,823.00	284,815.86	285,010.43
	Singletons (stdev)	69,889.22	69,906.60	69,862.20
After VQSR	Biallelic SNPs (average)	4,302,149.93	4,301,534.07	4,299,853.14
	Biallelic SNPs (stdev)	429,408.28	428,949.56	428,861.99
	Biallelic SNPs (min)	3,393,736	3,394,064	3,393,934
	Biallelic SNPs (max)	4,744,903	4,743,518	4,741,343
	Multiallelic SNPs (average)	28,690.14	28,664.71	28,645.43
	Multiallelic SNPs (stdev)	3028.99	3022.11	3019.16
	Singletons (SNPs, average)	247,511.64	247,212.57	247,270.29
	Singletons (SNPs, stdev)	62,155.76	61,953.47	61,967.99
	Filtered SNPs (average)	145,375.93	146,223.04	149,483.11
	Filtered SNPs (stdev)	46,580.10	46,955.26	48,246.66

Only SNPs are considered after VQSR

Since we do not have a *bona fide* true callset to compare our results to, we decided to consider “BP2019” as the callset to compare the other callsets to, hence all comparisons are relative to “BP2019” (if not specified otherwise). Before VQSR, the callset for “BP2019” consists of 20,301,167 biallelic SNPs, 85,510 multiallelic SNPs, 2,599,873 simple indels, 737,325 complex indels (see [45] for a definition of complex indels), and 7,975,044 singletons (variants appearing only once in the whole sample—depending on the type, these are a subset of the SNPs or indels) (Table 3). 3.32% of the biallelic SNPs and 5.70% of simple indels are absent from dbSNP v.151. Similar counts are obtained in “BP2015” and “3mask”; the largest difference is for the number of multiallelic SNPs in “3mask”, which is increased by 0.2514% compared to “BP2019” (i.e. count in “3mask” = 1.002514 * count in “BP2019”). “BP2015” and “3mask” have higher counts than “BP2019” for all but the number of singletons, where “BP2015” has a count decreased by 0.0025% compared to “BP2019”. The difference between “3mask” and “BP2019” is larger than between “3mask” and “BP2015”, except for the number of complex indels that shows the reverse tendency.

After running VQSR for SNPs, respectively 3.36% of biallelic SNPs and 11.94% of multiallelic SNPs are filtered out in “BP2019”. The corresponding percentages for “BP2015” are 3.47% and 12.14%, and for “3mask” they are 3.55% and 12.38%. Thus, the fraction of filtered SNPs is larger in “BP2015” and in “3mask” than in “BP2019”. This reverses the tendency of more SNPs in “BP2015” and “3mask” before VQSR: after VQSR, there are less bi- and multiallelic SNPs and less singletons in “BP2015” and “3mask” than in “BP2019”. In fact, “BP2015” has 3.37% more filtered variants and “3mask” 5.71% (these large differences with “BP2019” are a combination of less variants to start with in “BP2019” and a smaller proportion of filtered out variants in “BP2019”). After SNP VQSR, “3mask” has 0.14% less biallelic SNPs, 0.25% less multiallelic SNPs, and 0.10% less SNP singletons than “BP2019”; the corresponding percentages for “BP2015” are 0.11%, 0.22% and 0.12% less than “BP2019”. The proportion of biallelic SNPs absent from dbSNP v.151 decreases to 3.13% in “BP2019”.

We also looked at individual metrics (Table 3 and Additional file 3). On average in “BP2019”, an individual has 4,443,858 (stdev: 438,889.45) biallelic SNPs (by “biallelic SNPs” we mean than an individual’s genotype is different from homozygous reference, at a position with one alternative allele in the callset); 32,358 (stdev: 3,943.62) multiallelic SNPs (same definition as above except for positions with two or more alternative alleles in the callset); 508,132 (stdev: 46,623.55) simple indels; 346,818 (stdev: 25,452.05) complex indels; and 284,823 (stdev: 69,889.22) singletons. The individual with the highest number of biallelic SNPs (4,916,206) is a Ju|’hoansi (SGDPJUH1) while the individual with the lowest number of biallelic SNPs (3,442,414) is a French sample (HGDPFRE4). Similarly, it is always a French sample that has the lowest counts for multiallelic SNPs, simple and complex indels and singletons. A Khoe-San individual has the highest counts for multiallelic SNPs and simple indels; a Biaka individual (rainforest hunter-gatherer) has the highest count for the number of singletons; and, surprisingly, a non-African, the 1000GCEU2 individual (European ancestry from Utah, 1000 Genomes dataset) has the highest count of complex indels.

Comparing “3mask” and “BP2015” to “BP2019” we observed similar patterns for averages per individual as for the entire callset: in general, higher counts in “3mask” and “BP2015” (except for the count of multiallelic SNPs and singletons in “BP2015”), the largest difference being an increase of 0.2103% for the average number of complex indels in “3mask”. The increase in variants per individual in “3mask” compared to “BP2019” is significant for the five types of variants considered (one-sided paired *t*-test, p-values: 2*10^–12^ for biallelic SNPs, 1*10^–11^ for multiallelic SNPs, 2*10^–4^ for singletons, 3*10^–5^ for simple indels and 2*10^–7^ for complex alleles). The increase in “BP2015” compared to “BP2019” is significant for three types of variants (one-sided paired *t*-test, p-values: 3*10^–10^ for biallelic SNPs, 4*10^–5^ for simple indels and 2*10^–12^ for complex alleles); for multiallelic SNPs and singletons there is no significant difference. The SNP VQSR filter removes more variants in “3mask” and “BP2015” than in “BP2019”: 0.5827% more filtered SNPs in “BP2015” and 2.8252% more in “3mask”. Consequently, after SNP VQSR the average number of bi- and multiallelic SNPs and singletons are highest in “BP2019”: “3mask” has 0.0534% less biallelic SNPs, 0.1559% less multiallelic SNPs, and 0.10% less singletons than “BP2019”. The corresponding percentages for “BP2015” are 0.0143%, 0.0886%, and 0.12% (less than “BP2019”). The decrease in variants in “3mask” and “BP2015” compared to “BP2019” is significant (one-sided paired *t*-test, p-values: respectively 2*10^–10^ and 5*10^–4^ for biallelic SNPs, 2*10^–11^ and 6*10^–8^ for multiallelic SNPs, 4*10^–4^ and 9*10^–4^ for singletons).

**Fig. 2:**
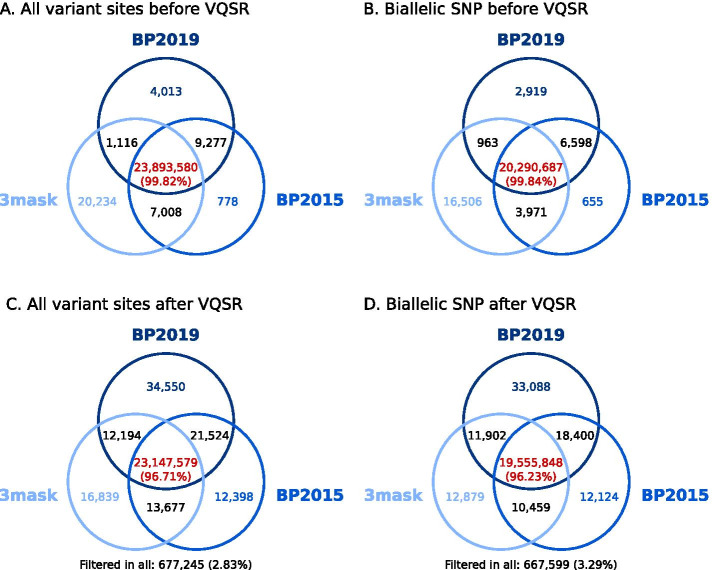
Most variants are common to “BP2019”, “BP2015” and “3mask”. Venn diagrams of the variants in “BP2019”, “BP2015” and “3mask” before VQSR (**A** and **B**) and after VQSR (**C** and **D**). The diagrams are not to scale. The percentages in parenthesis represent the percentage of all variants combined which are in the intersection. **A** All variant sites, before VQSR. **B** Biallelic SNPs, before VQSR. **C** All variant sites, after VQSR. **D** Bbiallelic SNPs, after VQSR

The similarities of counts for different features, for the entire callset and by individual, after three different ways of processing BAM files, suggest that the callsets are similar. We investigated this using GATK CombineVariants. Figure 2A and Additional file 4A show the partitioning of all variants (SNPs and indels). Before VQSR, the majority of the variants (99.82% of all variants combined) are identified by the three approaches. In particular, 99.94% of “BP2019” variants are in the intersection. The next largest fraction is variants found only in “3mask” (20,234 variants or 0.0845% of the combined variants). The pair of VCFs sharing most variants is “BP2015” and “BP2019”, followed by “3mask” and “BP2015”. The “3mask” approach results in the most private variants and “BP2015” the least. When summing all variants for each of the approaches—based on the CombineVariants output—we obtain higher counts than those reported in Table 3. This is due to complex variation, for example at the same position one of the VCFs has a SNP and the other has an indel. We verified the patterns described above by using GATK CombineVariants on the biallelic SNPs only (Fig. 2B, Additional file 4B). The same patterns are observed.

Finally, we performed the same analysis after VQSR. The results for all variants are in Fig. 2C and Additional file 4C, and for biallelic SNPs in Fig. 2D and Additional file 4D. Similar tendencies are observed for all variants and for biallelic SNPs. Considering biallelic SNPs, 96.23% of all variants are retained in the three VCFs after VQSR; 3.29% are removed from the three VCFs. The remaining 0.49% are variants found in only one VCF or variants found in two or three VCFs that have different filtering status. After VQSR, “BP2019vqsred” has almost three times more private biallelic SNPs (33,088) than “3maskvqsred” and “BP2015vqsred” (respectively 12,879 and 12,124). The same pair of VCFs than before VQSR share the most variants: “BP2015” and “BP2019”.

The overlap between the three pipelines is larger when restricting the analysis to regions of the genome accessible to short-read sequencing (1000 Genomes accessibility mask) (Additional file 5). Before VQSR (Additional file 5A, B), the results are qualitatively similar to those presented in Fig. 2 (e.g. most private variants in “3mask”). After VQSR (Additional file 5C, D), the tendencies are different, with roughly three times as many private variants in “3maskvqsred” and “BP2015vqsred” than in “BP2019vqsred”.

**Fig. 3:**
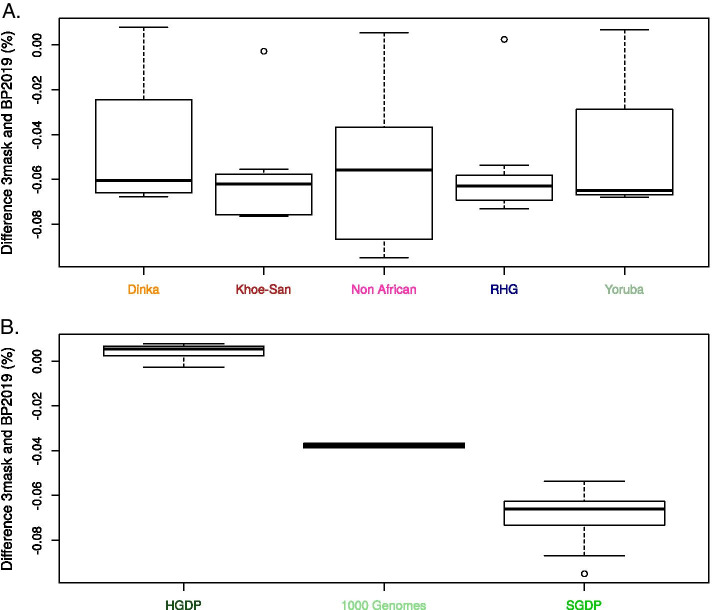
Differences in number of SNPs per individual are explained by dataset rather than ancestry. Boxplots of the difference between the number of SNPs per individual in “3maskvqsred” and “BP2019vqsred”, in percentage of “BP2019vqsred” (a negative percentage indicates more variants in “BP2019vqsred”). **A** Individuals are grouped by ancestry. **B** Individuals are grouped by dataset

We were interested in a possible effect of the population background (or ancestry) on the differences between the callsets. We plotted the difference in total number of SNPs by individual (kept after VQSR) between “3maskvqsred” and “BP2019vqsred”. Figure 3A shows the corresponding boxplots for each ancestry. The medians are similar in the five ancestries, and there is less variation in the Khoe-San and in the rainforest hunter-gatherers (RHG). When plotting according to dataset (Fig. 3B) the effect is much clearer. The difference between “3mask” and “BP2019” is smallest for the individuals from [44]-referred to as HGDP dataset- (average: + 0.004%), followed by the “1000 Genomes” two individuals (average: − 0.038%), and finally the individuals from the Simon Genome Diversity Project (SGDP) dataset (average: − 0.068%). Similar tendencies are observed for the difference in total number of indels by individual: the dataset impacts more the difference than the ancestry (Additional file 6). On the other hand, another metrics, the percentage of known variants (i.e. present in dbSNP v.151), seems to depend rather on the ancestry than on the dataset (Additional files 7, 8).

### The callset is impacted by the number of individuals at the joint genotyping step

One specificity of the GATK Best Practices is that the BAM pre-processing and the initial variant calling (HC) is run by individual. Only the joint genotyping step (GenotypeGVCFs) and downstream analyses (for example VQSR) are performed for the entire cohort at the same time. We compared the variant counts for our 28 individuals, first when the joint genotyping is done only for these 28 individuals (“3mask”), second when joint genotyping is done in a larger cohort (179 individuals) and the 28 individuals are extracted (“3mask + 28”, see Fig. 1). Note that this analysis was not done with the GATK Best Practices (i.e. not with “BP2019”). Metrics are reported in Table 4.

**Table 4:** Metrics in “3mask” and “3mask + 28”, at callset and individual level, before and after VQSR

	Metrics	3mask	3mask + 28
*Counts for the entire callset*			
Before VQSR	Biallelic SNPs	20,312,127	20,434,008
	Multiallelic SNPs	85,725	94,044
	*Total SNPs*	*20,397,852*	*20,528,052*
	Simple indels	2,601,657	2,564,122
	Complex indels	737,834	816,453
	*Total indels*	*3,339,491*	*3,380,575*
	Singletons	7,980,292	8,123,791
	Biallelic SNPs in dbSNP (%)	96.67%	96.67%
	Simple indels in dbSNP (%)	94.29%	94.21%
After VQSR	Biallelic SNPs	19,591,088	19,544,864
	Multiallelic SNPs	75,115	79,945
	*Total SNPs*	*19,666,203*	*19,624,809*
	Singletons (SNPs)	6,923,568	6,902,425
	Filtered SNPs	731,649	903,243
	Biallelic SNPs in dbSNP (%)	96.88%	96.96%
*Average (and standard deviation (stdev)) per individual*			
Before VQSR	Biallelic SNPs (average)	4,445,566.93	4,448,252.21
	Biallelic SNPs (stdev)	438,990.07	440,005.98
	Biallelic SNPs (min)	3,443,131.00	3,442,837.00
	Biallelic SNPs (max)	4,917,928.00	4,922,667.00
	Multiallelic SNPs (average)	32,414.75	33,932.54
	Multiallelic SNPs (stdev)	3961.22	4451.22
	*Total SNPs (average)*	*4,477,981.68*	*4,482,184.75*
	Simple indels (average)	508,597.68	490,154.68
	Simple indels (stdev)	46,465.10	46,446.32
	Complex indels (average)	347,547.21	370,585.18
	Complex indels (stdev)	25,401.04	30,851.51
	*Total indels (average)*	*856,144.89*	*860,739.86*
	Singletons (average)	285,010.43	290,135.39
	Singletons (stdev)	69,862.20	70,494.15
After VQSR	Biallelic SNPs (average)	4,299,853.14	4,305,202.11
	Biallelic SNPs (stdev)	428,861.99	430,653.45
	Biallelic SNPs (min)	3,393,934.00	3,394,319.00
	Biallelic SNPs (max)	4,741,343.00	4,749,854.00
	Multiallelic SNPs (average)	28,645.43	29,668.04
	Multiallelic SNPs (stdev)	3019.16	3267.90
	*Total SNPs (average)*	*4,328,498.57*	*4,334,870.14*
	Singletons (SNPs, average)	247,270.29	246,515.18
	Singletons (SNPs, stdev)	61,967.99	62,371.91
	Filtered SNPs (average)	149,483.11	147,314.61
	Filtered SNPs (stdev)	48,246.66	48,020.13

Only SNPs are considered after VQSR. Metrics names in italics have been calculated by the authors (i.e. not an output of Picard’s CollectVariantCallingMetrics)

Before VQSR, there are more variants in the “3mask + 28” callset than in the “3mask” callset (+ 0.60% for SNPs and + 1.23% for indels). This is also observed at the individual level, though to a smaller extent (+ 0.09% for SNPs and + 0.54% for indels). For SNPs, the increase is larger for multiallelic SNPs -i.e. SNPs that have more than one non-reference allele in the subset of 28 individuals- (for example before VQSR for the entire callset: + 0.60% for bi- and + 9.70% for multi-allelic SNPs). For indels on the other hand, the increase is due solely to more complex indels—there is a decrease in the proportion of simple indels. After SNP VQSR, we observed less biallelic SNPs in “3mask + 28” than in “3mask” at the callset level (− 0.23%). The number of multiallelic SNPs remains higher in “3mask + 28” (+ 6.43%). At the individual level, both the number of bi- and of multiallelic SNPs remain higher in “3mask + 28” (respectively 0.12% and 3.57%).

In the same way that we compared the variants in “BP2019”, “BP2015” and “3mask”, we investigated whether similar sets of variants were found in “3mask” and “3mask + 28”. Before filtering, 98.66% of the combined variants are called in the two VCFs. 1.06% are called only in “3mask + 28” and about four time less (0.28%) are called only in “3mask”. After SNP VQSR, 94.73% of the combined variants pass in the two callsets and 2.84% fail in the two callset. 1.46% of variants were found with both approaches but have different filtering outcomes.

Thus, it appears that in general, the two approaches call the same variants; with slightly more variants when there are 179 individuals at the joint genotyping step rather than 28. In particular, there is an increase of multiallelic SNPs and complex indels. However, for biallelic SNPs the picture changes after SNP VQSR at the callset level, with slightly less biallelic SNPs in “3mask + 28” than in “3mask” at the callset level.

For the SNPs kept after VQSR, some of the multiallelic variants in “3mask + 28” are biallelic in “3mask” (from 2.1% for chromosome 6 to 18.9% for chromosome 1, average when summing across chromosomes: 6.5%). These variants have a higher missingness than SNPs in general (18.15% average missingness, versus 1.44% average missingness for SNPs kept after VQSR in “3mask”). We investigated these sites more closely to determine which allele is called in place of the third allele in “3mask” (the reference or the first alternate allele). We looked at the number of alleles as a proxy for that, but we did not check the genotypes at an individual level. 6.1% of the variants have complex patterns, e.g. different numbers of alleles genotyped in the two VCFs (annotation “AN”) or three alternate alleles in “3mask + 28”. Another 84.4% of the variants have the reference allele called in “3mask” (for at least one of the second alternate allele copies); and the remainder, 9.5%, have the alternate allele called in “3mask”. The mean number of copies of the second alternate allele (in “3mask + 28”) did not differ significantly between the sites where the reference respectively the alternate is called (1.15 respectively 1.14 alleles, Student's *t*-test: *p* value 0.6126); nor did the mean number of genotyped alleles (46.69 respectively 46.27 alleles, Student's *t*-test: *p* value 0.4106). We conclude that there is a bias towards the reference allele at these sites, but note that these sites have higher than average missingness and are likely difficult to sequence, map or call.

The same approach could be applied to indels, though it is more complicated as the indels that differ between the two callsets are often in complex regions (for example with several indels in a row).

### Individual coverage might impact the number of variants

**Fig. 4:**
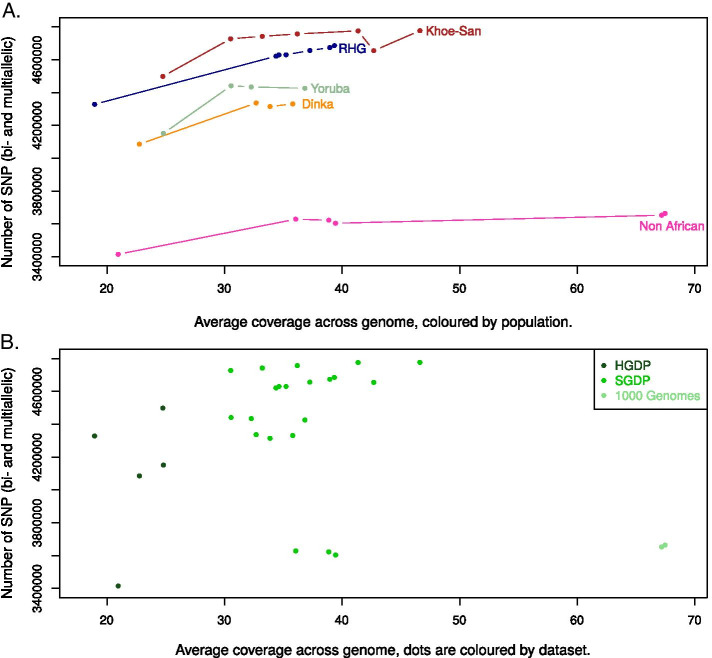
The total number of SNPs by individual is a function of coverage and ancestry. Total number of SNPs (bi- and multiallelic) per individual in “BP2019vqsred”. The y-axis starts at 3,400,000 SNPs. **A** Coloured by ancestry (the dots from a given ancestry are connected by lines). **B** Coloured by dataset

When possible, it is recommended to work with “high coverage” (or high depth) data. However, coverage can vary a lot between and within studies, which can potentially lead to biases. Here, we examined the correlation between individual coverage and number of SNPs (Fig. 4, Spearman’s rank correlation test, rho: 0.18, *p* value: 0.3576). We started by testing whether having an average depth above 30X (referred to as “> 30X”) or below or equal to 30X (referred to as “≤ 30X”) has an impact on the total number of SNPs after VQSR (for this we used “BP2019vqsred”). Five individuals, one from each of the populations, are in the “≤ 30X” category. The minimum coverage is ~ 19X. The difference in mean number of SNPs between the two groups is not significant (Wilcoxon rank-sum test, *p* value: 0.07112). As suggested by Fig. 4A, a confounding factor could be the population background: we know that the number of SNPs is greater in African than in non-African individuals. We performed the Wilcoxon rank-sum test in each population; the difference in mean number of SNPs between the two groups was not significant in any of the five populations.

**Fig. 5:**
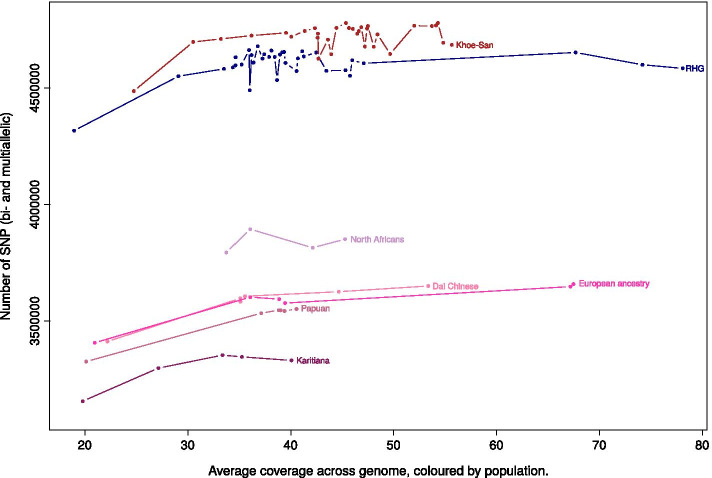
The total number of SNPs by individual in a larger dataset. Total number of SNPs (bi- and multiallelic) per individual in “3mask + vqsred”. The y-axis starts at 3,100,000 SNPs. Dots are coloured according to groups (the dots from a given group are connected by lines). The non-hunter-gatherer Sub-Saharan Africans are not shown as it is a very diverse group with respect to ancestry. RHG = Rainforest hunter-gatherers

Another limitation with “BP2019vqsred” could be the sample size. We performed the same test in a larger dataset: the “3mask + vqsred” dataset—same processing as in “3mask” but over 100 individuals at the joint genotyping step (Fig. 5, Spearman’s rank correlation test, rho: 0.3887117, *p* value: 9.713e-08). In this dataset, there is a significant correlation between coverage and number of SNPs called. The difference in mean number of SNPs between the “> 30X” and the “≤ 30X” samples is also significant (Wilcoxon rank-sum test, *p* value: 0.000136). There are two differences between “BP2019vqsred” and “3mask + vqsred”: number of individuals and processing. To rule out that the significance in “3mask + vqsred” and the non-significance in “BP2019vqsred” is due solely to the difference in processing, we did the same test in “3maskvqsred” (same processing as “3mask + vqsred” for the same set of individuals as “BP2019vqsred”); here the test is not significant (*p* value: 0.08204), i.e. the same as for “BP2019vqsred”. Thus it is more likely that the lack of significance of the tests in “BP2019vqsred” and “3maskvqsred” is due to the small sample size.

Another factor which impacts the number of variants is the population background. In “3mask + vqsred”, the proportion of individuals of non-African ancestry is larger in the “≤ 30X” group (0.42) than in the “> 30X” group (0.11). In order to limit the effect of the population background, we performed a Wilcoxon rank-sum test between the “> 30X” and “≤ 30X” groups considering individuals of African ancestry only. The number of SNPs is significantly different (greater) in the individuals with a coverage above 30X (*p* value: 0.007375). We note that the Wilcoxon rank-sum test with different coverage thresholds, for example 20X or 40X, are also significant (*p* value of 0.03323 and 1.461e−05 respectively). When we use a different metrics, the proportion of the genome with a depth of at least 15X, we observe a similar relationship with average genome coverage, but no effect of the ancestry (Additional file 9).

## Discussion

In this study, we undertake a comparison of pipelines based on the GATK Best Practices for Germline short variant discovery (SNPs + Indels) and using a realistic setting for a study of human genetic variation. We start by reviewing 29 studies of HTS data, focusing on their processing workflows, in particular the BAM processing, variant calling and callset refinement (Tables 1, 2). GATK tools are over-represented in this survey due to how the studies were selected. However, only few studies do include the three key steps of the Best Practices (BQSR, HC + GenotypeGVCFs and VQSR). This does not necessarily mean that these studies followed entirely the Best Practices, which include other steps as well, some not based on GATK tools (such as marking duplicates reads). Users might also choose different reference datasets than the ones recommended by GATK, or modify the behavior of tools in other ways. Moreover, the Best Practices change over time; for example, the indel realignment step is not recommended anymore. When citing the Best Practices, it would thus be informative to mention a date and to explicitly name which steps were included (and in the case of variant calling with GATK, whether UG or HC was used). This is why in this study, we prefer not to state whether a pipeline followed the Best Practices or not; rather we report whether different tools (with a focus on GATK) were used (Table 2). We also note that we could not identify all of the steps and/or softwares used for some of the published pipelines. More details are provided in Additional file 1. Overall, in order to enable easier comparisons between studies, HTS studies would benefit from more details in the description of their processing pipelines.

We applied several pipelines to the same set of genomes and compared the outcomes. One shortcoming of our study is that we do not have a “truth” set and have to resort to relative comparisons. We could have included genomes from the Genome in a Bottle Consortium [2, 46], for which reference material—including short variant calls—are available. One limitation is that such reference material is presently not available for Sub-Saharan African populations. Another option would have been to use SNP array results for the individuals included here (though the comparison is limited to ascertained variants in that case). Morever, this study uses GATK version 3, while the current version of GATK is version 4. However we think that our results should be transferable.

The three pipelines we compared differ in two steps: presence or absence of the indel realignment step, and recommended BQSR step versus custom BQSR step. Overall the callsets are very similar, though we observed that the Best Practices 2019 (no indel realignment and recommended BQSR) finds less variants than the other two pipelines before VQSR, while this tendency is reversed after VQSR. Concerning the indel realignment step, it is not surprising that the separate indel realignment step was abolished as it became obsolete after the introduction of the variant caller HC which performs local re-assembly of haplotypes and local indel realignment. Concerning the BQSR step, our concern was that the recommended procedure with the reference dataset dbSNP, where Sub-Saharan African variation is under-represented (although this is changing), would result in a loss of variation. However, this does not seem to be the case, as the callsets from the different approaches are very similar (in particular for SNPs after VQSR, and when restricting to the most accessible regions of the genome). We did not investigate in detail how the recommended versus the custom BQSR step impact base quality scores as the differences in the final callset (which is what interests us) were minimal. We did not observe clear effects associated to ancestry background, except that the variance of the difference between “3mask” and “BP2019” is smaller for the two groups of hunter-gatherers. This might be due to population structure for example. On the other hand, we did observe an effect of the dataset, particularly between “HGDP” and “SGDP”. The number of samples is much higher in the SGDP dataset (in this study) but the five different populations are represented at similar proportions in the datasets. The most obvious difference between these datasets is the average coverage (lower in HGDP).

The second comparison we conducted concerned one specific step of the pipeline: the joint genotyping step. We compared callsets for the same 28 individuals, where the joint genotyping was performed in only these 28 individuals or in a larger dataset of 179 individuals. We observed that callsets are overall very similar, but that more variants are found in the callset resulting from a larger number of individuals. However, this is true only for multiallelic SNPs after VQSR. If finding more variants is desired, it appears that including more individuals at the joint genotyping is an advantage—even if some of the individuals are not considered in downstream analyses. There is also an interplay between bi- and multiallelic SNPs, and possibly indels (when these different types of variants overlap, comparisons of callsets become complicated).

Finally, we looked into the correlation between coverage and number of variants. It is common to distinguish between “low coverage” (< 10X) and “high coverage” data. Exactly how much coverage is enough is unclear, and depends on the aim of the study; the choice of a sequencing depth is often a compromise with the sample size. We do observe in the larger dataset that coverage (> 30X or ≤  30X) correlates with the number of SNPs after VQSR, even when removing some of the signal which might be due to ancestry. This is a simple analysis and more data points (particularly in the range 25-35X) would be needed to issue more accurate recommendations; another possible analysis would be to downsample some of the higher coverage samples, and compare the number of variants found for different coverages. This was done for pigs (with a maximum coverage of 20X), where the authors recommend a depth of 10X [47]; in [48], the authors focus on detecting singletons in the context of human diseases and study the trade-off between depth of coverage and sensitivity. They conclude that a coverage of 15-20X is a good compromise between sample size and detection of singletons for association studies. As for the present study, the increase in number of SNPs as a function of coverage seems to level off at around 30–40X (Figs. 4, 5). This suggests that to capture as much of the variation as possible (for example to estimate heterozygosity as accurately as possible), at least 30–40X coverage is a good aim.

From our observations, we conclude that following the Best Practices (2019 version) for germline short variant discovery, with the GATK recommended datasets, does not limit the discovery of variants in Sub-Saharan African populations—at least when compared with other pipelines that use the same variant caller. This is an advantage as it is the fastest pipeline, it is well documented (compared to other less common pipelines), and does not require the user to assemble custom reference datasets. However we encourage the user to be cautious when using non-default options, such as working with all sites VCF (i.e. including non-variant sites), as they are less well documented (see the commands in Additional file 10 for an example). In particular, VQSR does not recalibrate non-variant sites, which means that variable sites are more likely to have been filtered out, creating a possible bias in downstream analyses. Most studies focus on variable sites only, but see [33] for a strategy to filter non-variable sites.

Regarding our concern of failing to call all of the true diversity present in the samples, comparing GATK tools to other variant callers would constitute an interesting study. This has been done in some studies [8, 16, 24], though often only the variants found by several approaches are kept—and variants found by a single variant caller are discarded. Thus, variants found by several variant callers were employed as a measure of accuracy. Another avenue to explore is the alternatives to mapping to a single reference genome, such as graph assemblies [49]. We also note that the present study focused mostly on SNPs, as they are the focus of many studies and are more easily tractable; however, it would be interesting to look more closely into the effect of different pipeline options on the quality of indel calling.

## Conclusions

We reviewed the processing pipelines of 29 HTS studies and found that while many studies used one or several GATK tools, few followed entirely the Best Practices and / or explicitly documented it. We compared several processing pipelines and found that following GATK 2019 Best Practices seems appropriate for populations of Sub-Saharan African ancestry. We also observed a correlation between average genome coverage and number of called variants. Taken together, this study allows us to make several recommendations, such as extensive documentation of HTS data processing, even when following GATK Best Practices; no obvious issues with following the Best Practices for underrepresented human populations; a large number of individuals at the joint genotyping step is preferred; and the average coverage matters, even above 20X coverage.

## Methods

### Literature review

We selected 29 studies using high coverage, high-throughput sequencing (HTS) data (23 focusing on humans, and six focusing on other mammals). These studies were selected by looking for papers citing one of the Genome Analysis Toolkit (GATK) articles (for example [10]). Several other articles were considered but not included, for example because we could not find enough information about the methods [50]; or because the pipeline was not comparable [51] (used Complete Genomics technology and pipelines). The included studies are summarized in Table 1.

We gathered information for different aspects of each study (Additional file 1). First we described the type of sample (species and in the case of human, population; sample size; sequencing platform). We then focused on the processing pipeline, which we divided into the following stages: steps prior to mapping (e.g. adapter removal) and mapping; information about the reference genome, such as build and inclusion of decoy sequences; BAM processing; variants calling; and callset recalibration. For each step, we reported (when information was available) the software used as well as the version of the software.

### Evaluation of BAM processing pipeline and of callset refinement strategies

#### Dataset assembly

We assembled a dataset of 28 individuals for which sequences are publicly available [8, 21, 44]. The type of data is Illumina paired-end short reads (Simon Genome Diversity Project (SGDP) [21]: 100 bp, HiSeq2000, insert length distribution 314 + − 20 bp; 1000 Genomes [8]: 250 bp, HiSeq2500 with modified chemistry; [44] (HGDP): 100 bp, HiSeq2000). Coverage is around 20X for the HGDP samples and ≥ 40X for the rest of the samples, with two samples (the two CEU samples from 1000 Genomes) > 60X. The individuals are distributed in five populations: six individuals with European background (two CEU, four French); four Yoruba; four Dinka; seven Khoe-San (five Ju|’hoansi, two #Khomani); and seven rainforest hunter-gatherers (two Biaka, five Mbuti). The data for [8, 21] was obtained from the EBI European Nucleotide Archive. The data for [44] was downloaded from (http://www.cbs.dtu.dk/suppl/malta/data/Published_genomes/bams/, not accessible anymore). The accessions numbers of the included individuals, their original IDs and IDs used in this study, as well as final coverage (duplicates removed) with processing pipeline “3mask”, are summarized in Additional file 2. Information about the number of reads, the number of mapped reads, and variant counts, are provided in Additional file 3.

We also performed some analyses on a larger dataset (179 individuals) comprising published [8, 21, 44, 52] and new unpublished human genomes. The proportions of the different human groups (non African; Khoe-San; rainforest hunter-gatherers; West African not hunter-gatherer; East African not hunter-gatherer) are similar in the two datasets.

### Generalities about processing and mapping

#### Reference files

The human reference genome (hg38) with decoy sequences was downloaded from the European Bioinformatics Institute (EBI 1000 Genomes GRCh38 reference genome) [53]. The reference was indexed with samtools/1.1 [54] (faidx), bwa/0.7.12 [6] (index) and picard/1.127 [55] (CreateSequenceDictionary).

A VCF file for dbSNP [40] version 144 was downloaded [56]. The chromosome names were changed to fit the notation in the reference genome. The file was then indexed with tabix/0.2.6 [57] (tabix). The same procedure was applied to more recent versions (dbSNP150 and dbSNP151). The version used at each step is specified in the detailed commands (Additional file 10).

For the VQSR, several resource datasets were downloaded on 2016-08-16 from the GATK beta bundle for hg38: a list of SNPs from phase 1 of 1000 Genomes; a high quality SNP callset from HapMap; and a set of SNPs produced by the Omni genotyping array.

The 1000 Genomes phase 3 accessibility mask was obtained to stratify variants (http://ftp.1000genomes.ebi.ac.uk/vol1/ftp/data_collections/1000_genomes_project/working/20160622_genome_mask_GRCh38/).

#### Mapping and duplicate marking

Detailed commands are provided in Additional file 10.

For mapping, we used bwakit/0.7.12 which is a package of scripts and binaries tailored for hg38. In particular, it deals with the “ALT contigs” and performs typing of the HLA regions. We used the mapping algorithm bwa mem [6]. The resulting BAM files were sorted and indexed with picard/1.126. The data from [44] was downloaded as mapped BAM. Thus, before mapping we reverted the mapped BAM to unmapped BAM with picard/1.126 RevertSam and then we shuffled and reverted the BAM to a FASTQ with samtools/1.1 [54] (bamshuf, bam2fq). The output of bam2fq is an interleaved FASTQ which was piped into the same mapping commands as for the rest of the samples.

In order to reduce the size of the BAM files we separated mapped and unmapped reads into two BAM files using samtools/1.1 (view). We went on processing the first file only.

Finally, we marked duplicates with picard/1.2.6 (MarkDuplicates).

### Processing of BAM: four processing pipelines

Detailed commands are provided in Additional file 10.

We compared three (plus one, see below) different pipelines for the processing of BAM. They are shown in Fig. 1. Briefly, the first pipeline—“BP2019”—corresponds to the 2019 “GATK Best Practices for Germline short variant discovery”. It contains a BQSR step with recommended reference dataset, a two steps variant calling step—HC and GenotypeGVCFs—and a callset refinement step, VQSR, which was run for SNPs only. Strictly speaking, the steps described in the previous section—mapping and marking duplicates—are also part of the GATK Best Practices.

The second pipeline—“BP2015”—has an extra step before the BQSR: indel realignment. It corresponds to the 2015 GATK Best Practices.

The third pipeline—“3mask”—has two extra steps: indel realignment as well as a variation of the BQSR step. We call this variation “triple mask BQSR” and it is described in [3]. In the recommended setting for BQSR, variants present in a reference dataset—for humans, dbSNP—are masked and variants not present in the reference dataset are recalibrated to obtain more accurate base quality scores. In the “triple mask BQSR”, we use dbSNP to mask, but also two VCFs obtained by calling variants on the sample itself, one after the default BQSR step, one without BQSR step (see Fig. 1 and Additional file 10). This is similar to the pipeline for organisms lacking reference datasets [34, 42, 43]. By masking with variants found in the sample itself, we hope to penalize less variation absent from the reference datasets.

Finally, the fourth pipeline—“3mask +”—is identical to the third pipeline except for the number of individuals at the joint genotyping (GATK’s GenotypeGVCFs) step—in the fourth pipeline there are 179 individuals. To be able to compare callsets across pipelines, we selected the 28 individuals from the large dataset directly after joint genotyping, using GATK’s SelectVariants with the trimAlternates option, and applied VQSR only to the subset (“3mask + 28”).

### Comparison of callsets

Picard/2.10.3 CollectVariantCallingMetrics counts variants in different categories (biallelic SNPs, multiallelic SNPs, indels, complex indels, singletons, filtered variants) and calculates some statistics (for example the percentage of variants present in a given dbSNP version). Count of variants are used for characterization and comparison of the different callsets.

GATK/3.7 CombineVariants was used to compare two or more callsets. We then used GATK/3.7 SelectVariants to generate the VCF files for the different sets.

VCFtools (version 0.1.13) [58] was used to analyze the VCF files, and in particular to extract annotations of interest.

Custom bash and Python (version 2.7.17) scripts were used to compare datasets; in particular a Python script was used to investigate variants multiallelic in one callset and biallelic in another.

R [59] was used to perform statistical tests, in particular Student’s *t*-test, Wilcoxon rank-sum test, and Spearman’s rank correlation coefficient.

## Supplementary Information

**Additional file 1.** Comparison of high coverage whole genome processing pipelines in 29 studies. Extended description of the high coverage whole genome processing pipelines summarized in Main Table 1

**Additional file 2.** Information about the individuals included in the processing pipelines comparison. Individuals IDs in this and original studies

**Additional file 3.** Quality control and variant counts (pipeline “BP2019”) by individuals and averaged by dataset or ancestry. Various quality control metrics relative to mapping, as well as variant counts—including percentage of variants in dbSNP v.151

**Additional file 4.** Overlap of the different pipelines—alternative representation. Alternative representation of the data in Fig. 2

**Additional file 5.** Most variants are common to “BP2019”, “BP2015” and “3mask” after applying an accessibility mask. Venn diagrams of the variants obtained by three processing pipelines, when restricting to the sites in the 1000 Genomes accessibility mask (Fig. 2 shows the results restricting the sites)

**Additional file 6.** Differences in number of indels per individual are explained by dataset rather than ancestry. Boxplots of the difference between the number of indels (simple and complex) per individual in “3mask” and “BP2019”, in percentage of “BP2019” (a positive percentage indicates more variants in “3mask”). A-Individuals are grouped by ancestry. B-Individuals are grouped by dataset

**Additional file 7.** Box plots of the percentage of known biallelic SNPs by individual, according to ancestry or dataset. Percentage of known biallelic SNPs (relative to dbSNP v.151) in “BP2019” (before VQSR). A-Individuals are grouped by ancestry. B-Individuals are grouped by dataset

**Additional file 8.** Box plots of the percentage of known simple indels by individual, according to ancestry or dataset. Percentage of known simple indels (relative to dbSNP v.151) in “BP2019” (before VQSR). A-Individuals are grouped by ancestry. B-Individuals are grouped by dataset

**Additional file 9.** Genome coverage is a function of average sequencing depth. Percentage of the genome covered by at least 15X per individual in “BP2019”, against the average sequencing depth. Dots are coloured by ancestry

**Additional file 10.** Commands used for the processing of BAM files. Commands used for the processing of the pipelines described in this study (see Fig. 1 for a graphical overview)

## Data Availability

The coverage and number of SNPs for the large dataset, as well as the genotypes for the subset “3mask +28”, are available from the authors upon reasonable request. The publicly available datasets analyzed during the current study are available through EBI (SGDP: European Nucleotide Archive, accession numbers PRJEB9586 and ERP010710 [21]); 1000 Genomes: FTP site, ftp://ftp.1000genomes.ebi.ac.uk/vol1/ftp/ [8]).

## Abbreviations

GATK: Genome Analysis Toolkit
SNP(s): single nucleotide polymorphism(s)
HTS: high-throughput sequencing
HC: HaplotypeCaller (GATK tool)
Best Practices workflow: GATK best practices for germline short variant discovery (SNPs + indels)
BQSR: Base Quality Score Recalibration (GATK tool)
VQSR: Variant Quality Score Recalibration (GATK tool)
UG: UnifiedGenotyper (GATK tool)
RHG: rainforest hunter-gatherers (from central Africa)
SGDP: Simon Genome Diversity Project
HGDP: refers to individuals from (44)
Hg38: human reference genome build 38 (also GRCh38)
CEU: Northern Europeans from Utah (1000 Genomes dataset
