# Supplementary material for: Comparison of sequencing data processing pipelines and application to underrepresented African human populations
Source: BMC Bioinformatics. 2021 Oct 9;22:488. doi: 10.1186/s12859-021-04407-x (PMC8502359; doi:10.1186/s12859-021-04407-x)
Supplement: Supplementary file 1 — Additional file 1. Comparison of high coverage whole genome processing pipelines in 29 studies. Extended description of the high coverage whole genome processing pipelines summarized in Main Table 1 [file 12859_2021_4407_MOESM1_ESM.pdf]

Comparison of sequencing data processing pipelines and application to underrepresented human populations Additional File 1.

Comparison of high coverage whole genome processing pipelines (humans)

Study	Samples details	Pre-processing + mapping	Human genome build	BAM processing	SNPs calling	Indels calling	Callset filtering	Comments
(Wong et al. 2013)	100 Southeast Asian Malays<br>Sequencing platform: Illumina HiSeq2000<br>~ 30X	-Alignment: Illumina CASAVA GERALD module	hg19 with PAR masked	-optical duplicate flagging, likely with the CASAVA pipelines<br>-exclusion of some individuals (ancestry; anomalous distribution of insert size)	Two methods:<br>1-single-sample variant caller in CASAVA,<br>2-SAMtools v0.1.17 multisample variant-caller mpileup	-Same as for SNPs<br>-For deletions > 50 bp: BreakDancer v.1.1 and VariationHunter v0.3	-Hard filtering	-
(H. L. Kim et al. 2014)	five Khoe-San<br>one Bantu-speaker<br>Sequencing platform: Illumina HiSeq	-bwa v.0.5.9 with default parameters except for '-q 15' to soft-trim low quality bases at the 3' end of reads	hg19	-GATK IndelRealignment<br>-Picard MarkDuplicates	-Obtained diploid consensus sequence with SAMtools mpileup -C 50<br>Then marked locations as missing data (see Callset filtering) and used SAMtools v0.1.18 to "identify the location of SNPs".	-Same as SNPs calling given the pipeline used (SAMtools).	-Hard filtering	-
(Auton et al. 2015)	High coverage genomes (30 individuals) from 1000 Genomes phase 3<br>Illumina HiSeq2500	-bwa mem<br>-Adapter clipping with MarkIlluminaAdapters.jar	GRCh37 with decoy	-Indel realignment GATK<br>-BQSR GATK	-NA	-NA	-NA	-
(Besenbacher et al. 2015)	30 Danes (ten trios)<br>Sequencing platform: Illumina HiSeq2000	-Adapter-Removal to:<br>trim the tails of the reads if the Phred quality dropped < 2.<br>collapse 180 bp insert size libraries into longer single end reads<br>-bwa-mem v.0.7.5.a	hg 19 decoy	-SAMtools v.0.1.19 and Picard v.1.96 to "process the alignment files and mark duplicate reads" (at lane level).<br>-Indel realignment with GATK v.2.7.2 (at lane level and after merging) with Mills and 1000G gold standard and dbSNP138.<br>-BQSR with GATK v.2.7.2 at lane level and with	-GATK v2.7.2: HaplotypeCaller for multi-sample genotyping	-Same as for SNPs.	GATK: VQSR<br>-SNPs:<br>datasets: HapMap, Omnichip, 1000G phase1 high confidence variants, dbSNP138<br>Annotations: QD, MQRankSum, ReadPosRankSum, FS, DP<br>--numBadVariants	-

				dbSNP138.			5000<br>tranche: 99.5<br>-INDELS:<br>dataset: Mills and 1000G<br>--maxGaussians 4<br>--numBadVariants 1000<br>tranche: 95.0	
(Gudbjartsson et al. 2015)	2,636 Icelanders at ~ 20 X<br>Sequencing platform: Illumina GAII$_x$ and/or HiSeq2000	-BWA v.0.5.7-0.5.9 then alignments merged into a single BAM	hg18	-Alignments for a given individual merged into a single BAM file<br>-Picard v.1.55: duplicates marking<br>-GATK v.1.2-29-g0acaf2d: realignment around indels and BQSR	-Multi-sample variant calling GATK v.2.3.9 (algorithm: NA, but likely UnifiedGenotyper)	-Multi-sample variant calling GATK v.2.3.9 (algorithm: NA, but likely UnifiedGenotyper)	-Hard filtering for SNVs and indels, using GATK recommended filters and additional filters.<br>-Regional filter (simple-repeat regions).	-
(Nagasaki et al. 2015)	1,070 Japanese<br>Sequencing platform: HiSeq 2500	Two strategies:<br>1-Bowtie2 v.2.1.0 with '-X 2000' option<br>2-bwa-mem v.0.7.5a-r405 with default option	hg19 decoy		Depending on the mapping strategy:<br>1-BCFtools v.0.1.17-dev (Bowtie alignment)<br>2-GATK v2.5-2 (bwa alignment) (algorithm: NA)<br><br>For downstream analyses, used the outcome of the first strategy (bowtie+BCFtools).	-Short structural variants (<100 bp): GATK HaplotypeCaller v.2.5-2 on bwa alignment<br>-Long structural variants: BreakDancer v.1.1 and Pindel v.0.2.5a3<br>-CNV: CNVnator v.0.3	-Hard filtering (genotype depth filter per individual, SNV depth filter per locus, genome complexity filter, tool bias filter, population genetics filter).	-
(UK10K Consortium et al. 2015)	~10,000 individuals of which:<br>~4,000: WG at ~7X from two British cohorts of European ancestry and<br>~6,000: exomes at ~80X<br>Sequencing platform: Illumina HiSeq	-sequencing reads failing QC removed with the Illumina GA pipeline<br>-bwa v.0.5.9-r16: bwa aln -q15	hg19	-Obtained sorted BAM from SAM files: Picard v1.36 and SAMtools v0.1.11 / or SAMtools v0.1.8 only.<br>-Mark duplicates with Picard MarkDuplicates / or SAMtools rmdup<br>-GATK v.1.1-5-g6f43284, Picard v.1.64, SAMtools v.0.1.16: Realignment around indels, BQSR, addition of BAQ tags, merging and	-SAMtools mpileup -C50 -d 8000/bcftools v. 0.1.18-r579 (pooling all individuals).<br>-GATK v.1.3-21 UnifiedGenotyper for SNVs to recall the sites found by the SAMtools/BCFtools pipeline to generate	-SAMtools mpileup -C50 -d 8000/bcftools v. 0.1.18-r579 (pooling all individuals).	-SNV: GATK VQSR with default parameters and datasets (dbSNP132), tranche 99.5<br>-indels: pre-filtering due to technical issues + GATK VQSR, did not use dbSNP but used 1000 Genomes	-

				duplicate marking (“follows that used for Illumina low-coverage data in 1000GP”.)	annotations used in VQSR.		Phase I.	
(Fakhro et al. 2016)	108 unrelated Qataris<br>Sequencing platform: Illumina HiSeq2500<br>(+ 1,268 exomes on one of three platforms)<br>One individual sequenced on three platforms + HiSeqX	-BWA v.0.5.9 with maximum insert size 3 kb	hg19	-SAMtools: PCR duplicates removal<br>-“mapped reads were prepared for variant calling using GATK Best Practices”: GATK realignment of indels and BQSR?	-Group-call with GATK (algorithm and version: NA, but likely UnifiedGenotyper - see Comments)	-Short indels (<300 bp) called with the CASAVA v1.9 pipeline by Illumina	-Hard filtering based on comparing different batches and results for the quadruple-sequenced Qatari.	-Also tested mapping to a Qatari reference genome.<br>-Joint calling of exomes and genomes with GATK UnifiedGenotyper
(Haber et al. 2016)	four Lebaneses<br>eleven Chadians<br>four Greeks<br>Sequencing platform: Illumina HiSeq X or HiSeq2500<br>Genomes were not the main focus of the paper.	NA	NA	NA	-SAMtools v.1.2 and BCFtools v.1.2: samtools mpileup -q 20 -Q 20 -C 50 | bcftools call -c -V indels	-Same as SNPs calling given the pipeline used (SAMtools + BCFtools)	NA	-
(Malaspinas et al. 2016)	83 Australians<br>25 Papuans<br>Sequencing platform: Illumina HiSeq X	-adapter trimming (Adapter-Removal-1.5.4) + trimmed leading / trailing stretches of Ns + bases with quality 2. Kept reads of >= 30 bp<br>-bwa-mem<br>-re-aligned with stampy v.1.0.23 (option: keep good mapped reads from bwa)	hg19	-Picard v.1.127<br>-add read groups<br>-sort reads<br>-merge reads at the library level<br>-duplicate reads marked and merged at the sample level<br>-GATK: indel realignment (with Mills and 1000G gold standard)<br>-Removed reads with mapping quality <30<br>-SAMtools calmd: recalculated the md tags and added extended BAQs	-Per sample: SAMtools v.0.1.18 and BCFtools v.0.1.17: mpileup -C50 | bcftools	-Same as SNPs calling given the pipeline used (SAMtools + BCFtools)	-Hard filtering	-
(Mallick et al. 2016)	300 samples from 142 worldwide populations (22 of which were produced earlier)<br>Sequencing platform: Illumina HiSeq2000	-adapter trimming: extract and shuffle reads from BAM with htslib, run trimadapt<br>-bwa-mem v0.7.10-r1005-dirty<br>-RG added during alignment	hg19 decoy	-mark optical duplicates with *samblaster*<br>-SAMtools: sort	-Per sample. Biallelic SNPs. Modification of UnifiedGenotyper developed with the GATK team to minimize reference bias.	-FermiKit-0.8 for the 263 fully public samples.	-Hard filtering	-

(Telenti et al. 2016)	10,000 individuals: “representatives of major human populations and ancestries” (8,096 unrelated)<br>Sequencing platform: Illumina HiSeqX	-ISIS Analysis Software v.2.5.26.13	hg38 with Y chr PAR masked	-ISIS Isaac Aligner v. 1.14.02.06: mark duplicates.<br>-Picard v. 1.113-1.131: to “characterize[d]” bam files.	-ISIS Isaac Variant Caller v.2.0.17 with default settings.	-ISIS Isaac Variant Caller v.2.0.17 with default settings.	-Comparison of variants found in NA12878 to the results from the Genome in a Bottle Consortium.	-
(Ameur et al. 2017)	1000 Swedes	-bwa mem -M -R (added read group information)	hg19	-SAMtools: sort alignment<br>-Picard: merge all BAM files corresponding to a sample<br>-GATK IndelRealignment with Mills and 1000G gold standard and dbSNP138<br>-Picard MarkDuplicates<br>-GATK BQSR with dbSNP138	-HaplotypeCaller<br>CombineGVCF<br>GenotypeGVCFs<br>CatVariants (merge the non-overlapping intervals)	-Same as SNPs calling given the pipeline used.	-VQSR (SNPs and indels) GATK-recommended datasets	-
(Choudhury et al. 2017)	24 South Africans:<br>-eight Coloured<br>-seven Sotho-speakers<br>-eight Xhosa-speakers (Nguni)<br>-one Zulu-speaker (Nguni)<br>Sequencing platform: Illumina HiSeq2000	-Isaac Analysis Pipeline v.2.0.2. During mapping, low quality 3’ ends and of adaptor sequences were trimmed.	hg19	-Isaac Analysis Pipeline v.2.0.2: marking of duplicates, realignment of indels.<br>-GATK v.3.2.2: BQSR.<br>-SAMtools v.1.1-26-g29b0367: flagstats.<br>-Checked that all samples pass thresholds (quality of reads).	Three callsets:<br>1.Isaac variant caller<br>2.GATK v.3.2-2 HaplotypeCaller default parameters<br>3.GATK v.3.2-2 HaplotypeCaller stand_call_conf 50<br>For approaches 2 and 3: GenotypeGVCFs for the three groups (Coloured, Sotho, Xhosa+Zulu) indepently.	-Isaac variant caller (indels and CNV)	For SNV:<br>> Isaac callset: hard filtering<br>> GATK callsets: VQSR with default parameters and datasets, tranche 99.5<br>Then took overlap.	-
(Harris et al. 2018)	150 individuals from Peru (Native American and mestizo) ~35X Illumina HiSeqX10	-bwa mem	Hg19	-MarkDuplicates Picard<br>-IndelRealignment GATK<br>-BQSR GATK	-HaplotypeCaller GATK<br>-GenotypeGVCF GATK<br>It is written “variants called using GATK in each individual’s genome”. UnifiedGenotyper is run too.	-See for SNPs	-Hard filtering	Processing was performed at the sequencing center. Do not specify version. www.nygenome.org/wp-content/uploads/2018/01/Whole-Genome-Sequencing-Germline.pdf

(J. Kim et al. 2018)	50 unrelated Korean individuals	-cleaning of raw reads by Sickle: keep quality score >20 and read length >50bp<br>-bwa	hg19	-Removal of PCR duplicates<br>-Indels realignment and recalibration (software: NA)	-GATK-Lite-2.3-9: UnifiedGenotyper by individual	-GATK-Lite-2.3-9: UnifiedGenotyper by individual	-Hard filtering for SNVs and indels.	-
(Jeong et al. 2018)	17 individuals (four Tibetans mother-daughter duos and three Sherpa trios), ~20X (plus 36 low coverage).<br>Illumina HiSeq2500 and 4000	-bwa backtrack v0.7.4 with -q 15 option	hg19	-MarkDuplicates with Picard v1.98<br>-IndelRealignment GATK v2.8-1<br>-BQSR GATK v2.8-1<br>-filtered reads with Phred-scaled mapping quality lower than 30 with SAMtools v1.2	-GotCloud	-GotCloud	-GotCloud	-
(Natarajan et al. 2018)	“TOPMED phase 1” cohort: 8,394 individuals from the USA (various ancestries, three studies: JHS, FHS, OOA)	-Not specified	1000 Genomes hs37d5 build 37 decoy reference sequence	-TOPMED phase 1: GotCloud	-TOPMED phase 1: GotCloud “freeze 3a” (vt discover2 software)	-TOPMED phase 1: GotCloud “freeze 3a”	-TOPMED phase 1: GotCloud “freeze 3a” (hard filtering)	-
	“TOPMED phase 2” (MESA study, 4510 individuals) and FINRISK (n=1165) and EST (n=2255)<br>Illumina HiSeqX		hg19	-Not specified	-HaplotypeCaller GATK v3<br>-GenotypeGVCF	-HaplotypeCaller GATK v3<br>-GenotypeGVCF	-VQSR GATK<br>-hard filtering	-
(Okada et al. 2018)	2234 individuals from Japan<br>20-35X<br>Illumina HiSeq2500 or Illumina HiSeq X Five	-Adaptor trimming with Trimmomatic (v0.36 for datasets 2 and 3)<br>-bwa mem v0.7.5a	GRCh37/ hg19, hs37d5 - decoy	-MarkDuplicates Picard (v1.106 for datasets 1 and 2, v2.5.0 for dataset 3)<br>-Indel realignment and BQSR GATK (v3.2-2, 3.5-0 and 3.6 respectively)	-HaplotypeCaller GATK<br>-GenotypeGVCF GATK (?)	-Same as for SNPs	-Hard filtering<br>-then VQSR GATK<br>-genotype refinement with beagle<br>-and more...	-
(S. Fan et al. 2019)	43 individuals from 22 African groups (analyzed together with 29 previously sequenced – Mallick 2016).<br>Illumina HiSeq2000	-Adaptor trimming<br>-bwa mem version 0.7.12	Hs37d5	-mark optical duplicates with *samblaster*<br>-SAMtools: sort	Per sample. Biallelic SNPs. Modification of UnifiedGenotyper developed with the GATK team for GATK UnifiedGenotyper to minimize reference bias.	-NA	-Hard filtering	Same as (Mallick et al. 2016)
(Lorente-	Nine individuals from	-bwa v0.6.1 aln -n 6 -q	GRCh37	-MarkDuplicates Picard v1.70	-UnifiedGenotyper	-Not called	-VQSR GATK v2.5-	-

Galdos et al. 2019)	African origin.<br>21-47X<br>Illumina HiSeq2000	15 and sampe	with Epstein-Barr virus	-IndelRealignment GATK v2.5-2<br>-BQSR GATK v2.5-2	GATK v2.5-2		2<br>-Defined a callable genome filter (based on their data and other information e.g. TandemRepeatMarker)	
(Serra-Vidal et al. 2019)	21 male individuals (17 North Africans, 2 Basque and 2 Iraqi)<br>~26X<br>Illumina HiSeq2000	-Assess read quality with fastqc<br>-bwa v0.7.7	hg19	-Merged mapped reads<br>-MarkDuplicates Picard v2.8.3<br>-IndelRealignment GATK v4.0.12<br>-BQSR GATK v4.0.12	-UnifiedGenotyper GATK GATK v4.0.12	-Not called	-VQSR GATK GATK v4.0.12<br>-Defined a callable genome filter (based on their data and other information e.g. TandemRepeatMarker)	-
(Bergström et al. 2019)	787 individuals fro 54 populations (analyzed with 142 previously sequenced – Mallick 2016, Meyer 2012, Raghavan 2015)<br>of which 26: linked-read technology (physical phasing).<br>Illumina HiSeqX ~35X	-Adaptor trimming with bamadapterclip (biobambam package)<br>-Mapping with bwa mem v0.7.12 with the -T 0 parameter<br>-Marking of duplicates with bamstreamingmarkduplicates (biobambam package).	GRCh38 with decoy	-None.	-HaplotypeCaller version 3.5.0. genotype priors without bias towards the reference allele through the “--input-prior 0.001 –input-prior 0.4995” arguments; “--pcr_indel_model NON” for the PCR-free libraries; “--includeNonVariantSites”.<br>-GenotypeGCVF (guessed from the text).	-Same as for SNPs.	-Hard filtering because they wanted to filter both variant and non-variant sites (VQSR: only for variant sites). Filter on GQ/RGQ and DP.<br>-VQSR<br>-excess heterozygosity with BCFtools fill-tags plugin.	Wellcome Sanger Institute sequencing facility automated pipeline

Comparison of high coverage whole genome processing pipelines (non-humans)

Study	Samples details	Pre-processing + mapping	reference	BAM processing	SNPs calling	Indels calling	Variant recalibration	Comments
(Z. Fan et al. 2014)	one Tibetan macaque analyzed together with four comparative genomes<br>Sequencing platform: Illumina HiSeq2000	-Bowtie2 under local alignment algorithm with very sensitive model	rheMac2 (Indian rhesus macaque)	-Picard: mark duplicate reads<br>-GATK: indel realignment followed by fixing the mate pair information with FixMateInformation.jar<br>-GATK: “empirical BQSR”. Started by doing a variant call on the Tibetan macaque to get a list of SNPs to exclude from BQSR (to replace the dbSNP dataset used for humans).	-GATK: UnifiedGenotyper (each sample separately). Standard minimum confidence thresholds to 0.	-Same as SNPs calling.	-Hard filtering.	-
(Z. Fan et al. 2016)	nine wolves<br>one coyote<br>one golden jackal<br>analyzed together with 23 comparative canine genomes<br>Sequencing platform: Illumina HiSeq2000	-Bowtie2 under local alignment algorithm with very sensitive model	CanFam3 .1	-Picard: mark duplicate reads<br>-GATK: indel realignment followed by fixing the mate pair information with FixMateInformation.jar<br>-GATK: “empirical BQSR”. Started by doing a variant call on the Tibetan macaque to get a list of SNPs to exclude from BQSR (to replace the dbSNP dataset used for humans).	-GATK: UnifiedGenotyper (each sample separately). Standard minimum confidence thresholds to 0.	-Same as SNPs calling.	-Hard filtering.	-Same as (Z. Fan et al. 2014). The same pipeline was also used in studies not reviewed here (Zhang et al. 2014; Freedman et al. 2014)
(Friedenberg , Meurs, and Mackay 2016)	64 dogs across eight breeds<br>Sequencing platform: Illumina HiSeq 2000 or 2500	-Trimmomatic v0.32: trim reads<br>-bwa v.0.7.10	canFam3	-Picard v.1.115 (analysis performed not specified – mark duplicates?)<br>-GATK v.3.4: BQSR and indel realignment	-GATK: HaplotypeCaller	-GATK: HaplotypeCaller	-VQSR:<br>-dbSNP139<br>-Illumina CanineHD BeadChip<br>-SNPs: tranche 99.9, indels: 99	-
(Friedenberg , Lunn, and Meurs 2017)	20 dogs (Standard Poodles)	-Trimmomatic v0.32: trim reads<br>-bwa v.0.7.10	canFam3	-Picard v.1.115 (analysis performed not specified – mark duplicates?)<br>-GATK v.3.4: BQSR and indel realignment	-GATK: HaplotypeCaller	-GATK: HaplotypeCaller	-VQSR:<br>-dbSNP139<br>-Illumina CanineHD BeadChip<br>-SNPs: tranche 99.9, indels: 99	-Same as (Friedenberg, Meurs, and Mackay 2016)
(Bimber et	21 rhesus macaques	-Trimmomatic adaptive	MacaM	-GATK: indel realignment	-GATK:	-Same as SNPs	-Hard filtering	-

al. 2017)	including two trios and multiple parent/child pairs<br>Sequencing platform: HiSeq2000 or 3000	quality trimming<br>-bwamem		-Picard: marking of duplicates	HaplotypeCaller + GenotypeGVCFs (emitting and calling threshold: 20)	calling		
(Pfeifer 2017)	seven publicly available green monkey (three generations pedigree)<br>Sequencing platform: Illumina HiSeq2000	-bwa-mem v.0.7.13	Repeat masked *Chlorocebus sabaeus* (green monkey) v.1.1 + Epstein-Barr virus	-Per read group:<br>-Picard v.2.1.1: Marking duplicates<br>-GATK v.3.5: indel realignment<br>-GATK v3.5: BQSR with ~500 k variants from the genome-wide SNPs panel of the Vervet Genetic Mapping Project<br>-Per sample:<br>-Picard v.2.1.1: Marking duplicates	-GATK v3.5: HaplotypeCaller, default heterozygosity rate 0.001 + GenotypeGVCF: 1-single-sample, 2-joint genotyping.	-Same as SNPs calling	-Hard filtering	-
