# Supplementary material for: Comparison of sequencing data processing pipelines and application to underrepresented African human populations
Source: BMC Bioinformatics. 2021 Oct 9;22:488. doi: 10.1186/s12859-021-04407-x (PMC8502359; doi:10.1186/s12859-021-04407-x)
Supplement: Supplementary file 4 — Additional file 4. Overlap of the different pipelines—alternative representation. Alternative representation of the data in Fig. 2 [file 12859_2021_4407_MOESM4_ESM.pdf]

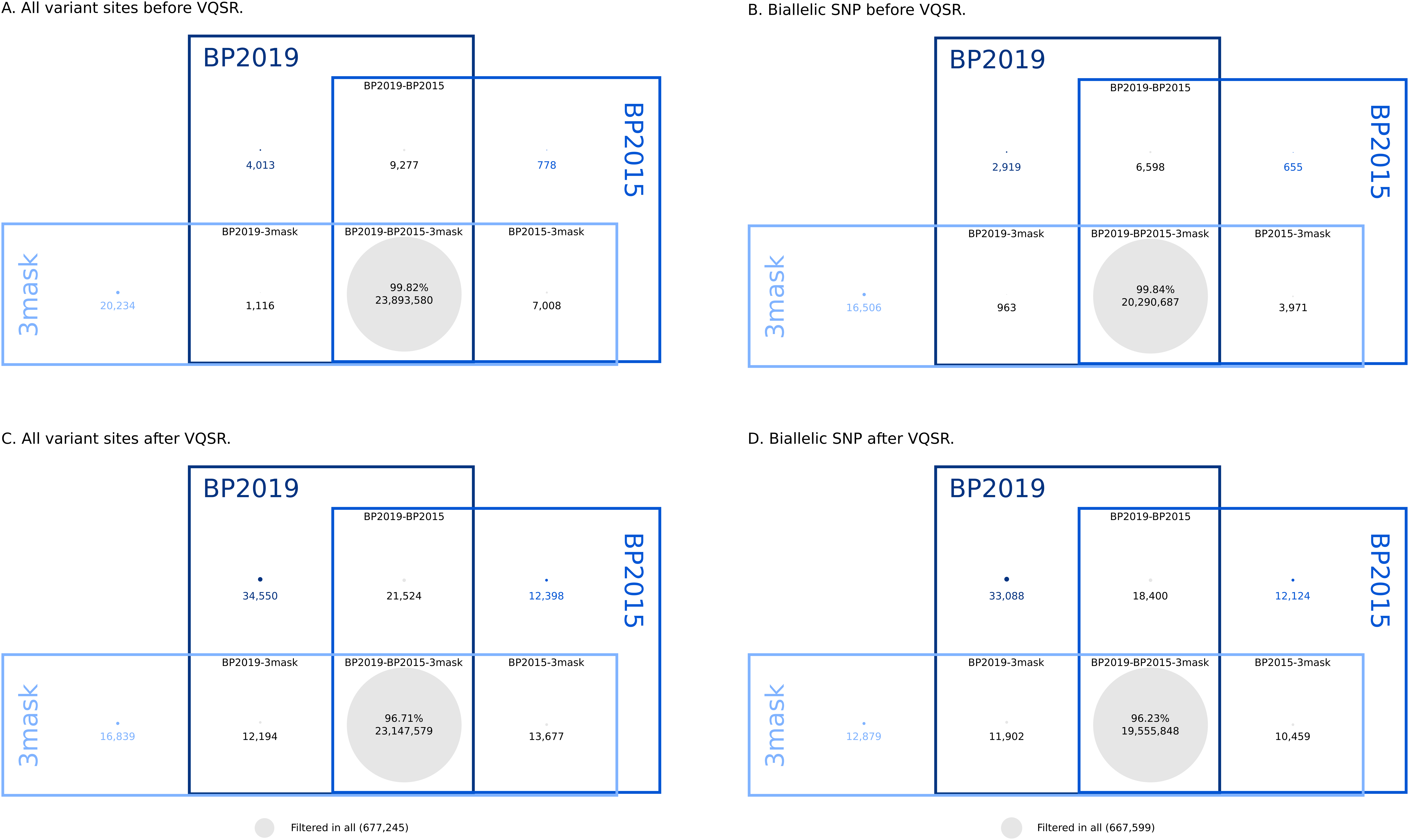
A. All variant sites before VQSR.
BP2019
BP2015
3mask
BP2019-BP2015
BP2019-3mask
BP2019-BP2015-3mask
BP2015-3mask
4,013
9,277
778
20,234
1,116
99.82%
23,893,580
7,008
B. Biallelic SNP before VQSR.
BP2019
BP2015
3mask
BP2019-BP2015
BP2019-3mask
BP2019-BP2015-3mask
BP2015-3mask
2,919
6,598
655
16,506
963
99.84%
20,290,687
3,971
C. All variant sites after VQSR.
BP2019
BP2015
3mask
BP2019-BP2015
BP2019-3mask
BP2019-BP2015-3mask
BP2015-3mask
34,550
21,524
12,398
16,839
12,194
96.71%
23,147,579
13,677
Filtered in all (677,245)
D. Biallelic SNP after VQSR.
BP2019
BP2015
3mask
BP2019-BP2015
BP2019-3mask
BP2019-BP2015-3mask
BP2015-3mask
33,088
18,400
12,124
12,879
11,902
96.23%
19,555,848
10,459
Filtered in all (667,599)
