# Supplementary material for: Comparison of sequencing data processing pipelines and application to underrepresented African human populations
Source: BMC Bioinformatics. 2021 Oct 9;22:488. doi: 10.1186/s12859-021-04407-x (PMC8502359; doi:10.1186/s12859-021-04407-x)
Supplement: Supplementary file 5 — Additional file 5. Most variants are common to “BP2019”, “BP2015” and “3mask” after applying an accessibility mask. Venn diagrams of the variants obtained by three processing pipelines, when restricting to the sites in the 1000 Genomes accessibility mask (Fig. 2 shows the results restricting the sites) [file 12859_2021_4407_MOESM5_ESM.pdf]

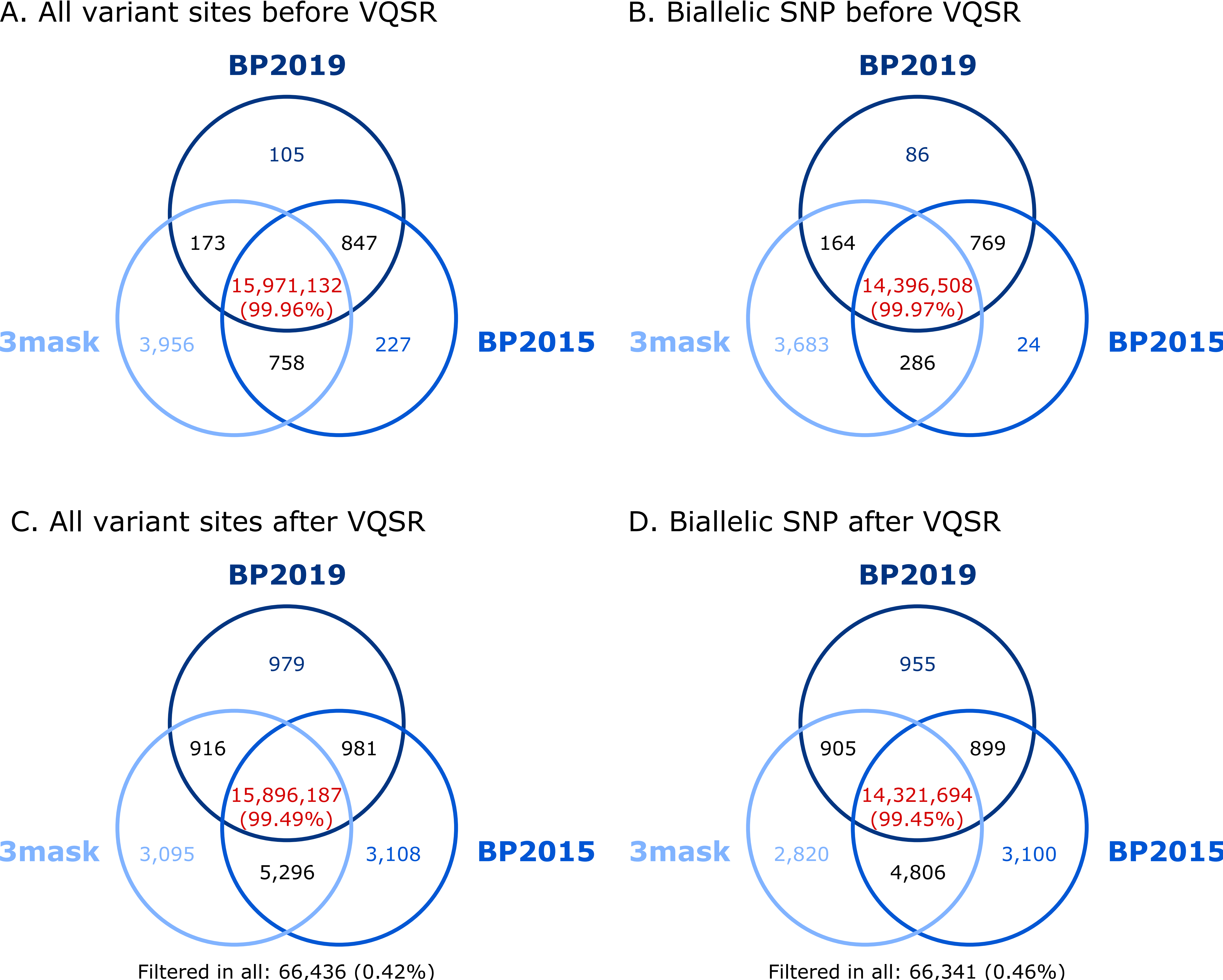
A. All variant sites before VQSR
BP2019
105
173
847
15,971,132
(99.96%)
3mask
3,956
758
227
BP2015
B. Biallelic SNP before VQSR
BP2019
86
164
769
14,396,508
(99.97%)
3mask
3,683
286
24
BP2015
C. All variant sites after VQSR
BP2019
979
916
981
15,896,187
(99.49%)
3mask
3,095
5,296
3,108
BP2015
Filtered in all: 66,436 (0.42%)
D. Biallelic SNP after VQSR
BP2019
955
905
899
14,321,694
(99.45%)
3mask
2,820
4,806
3,100
BP2015
Filtered in all: 66,341 (0.46%)
