# Supplementary figures and images for: Comparison of sequencing data processing pipelines and application to underrepresented African human populations
Source: BMC Bioinformatics. 2021 Oct 9;22:488. doi: 10.1186/s12859-021-04407-x (PMC8502359; doi:10.1186/s12859-021-04407-x)

**A.****Difference between 3mask and BP2019 in percentage of BP2019**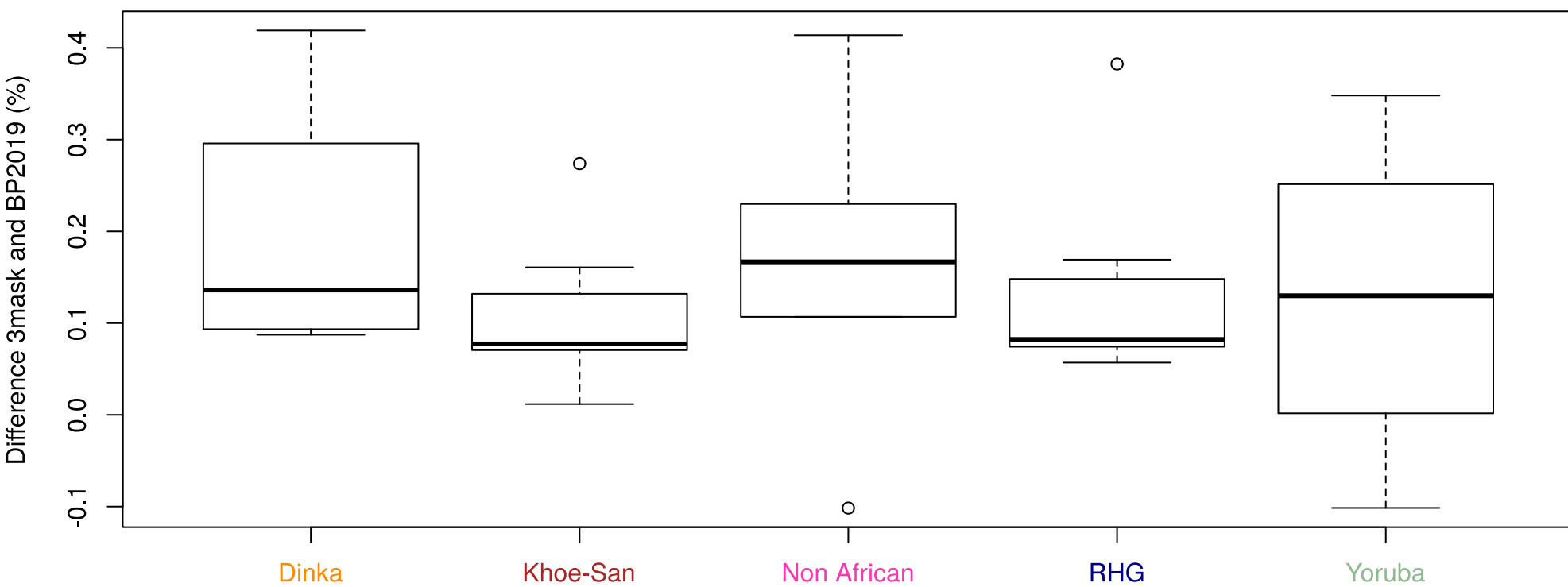**B.**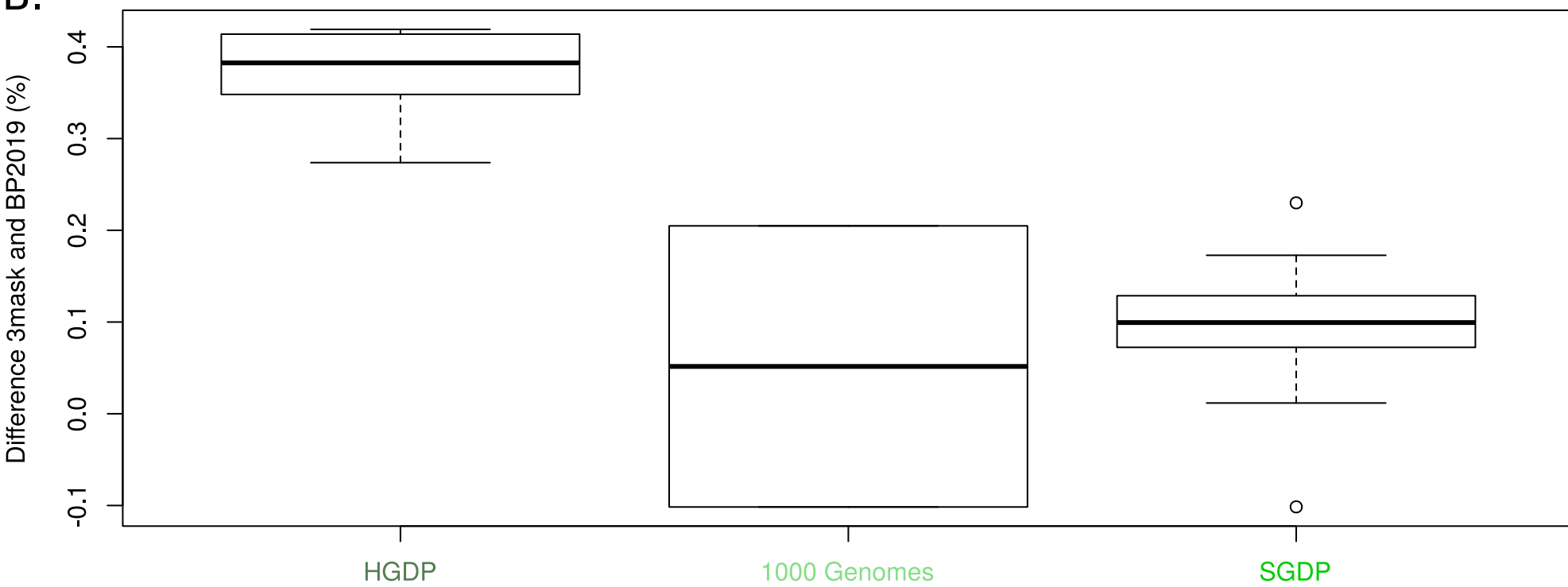

Supplement: Supplementary file 6 — Additional file 6. Differences in number of indels per individual are explained by dataset rather than ancestry. Boxplots of the difference between the number of indels (simple and complex) per individual in “3mask” and “BP2019”, in percentage of “BP2019” (a positive percentage indicates more variants in “3mask”). A-Individuals are grouped by ancestry. B-Individuals are grouped by dataset [file 12859_2021_4407_MOESM6_ESM.pdf]

# Proportion of biallelic SNPs in dbSNP v.151

A.

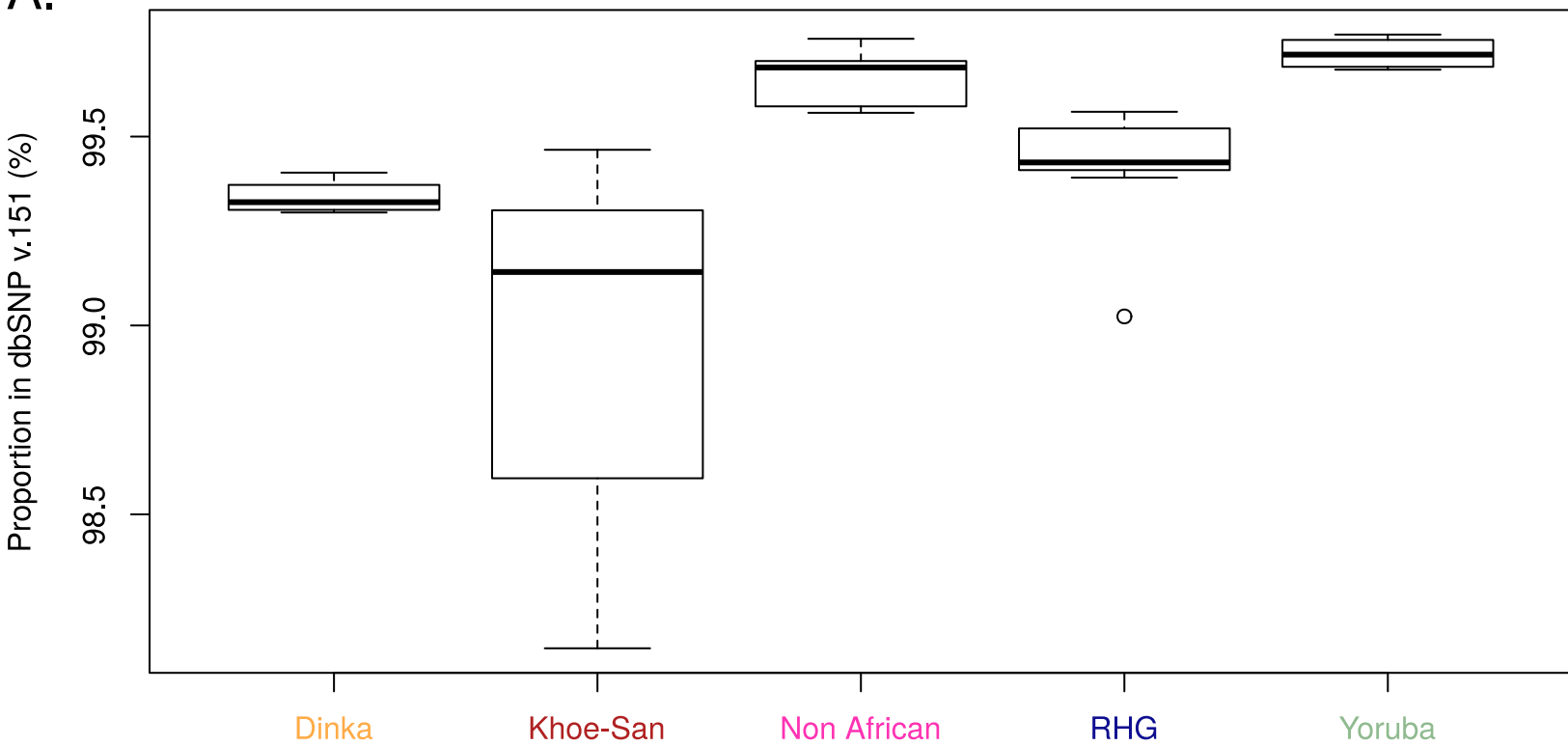

B.

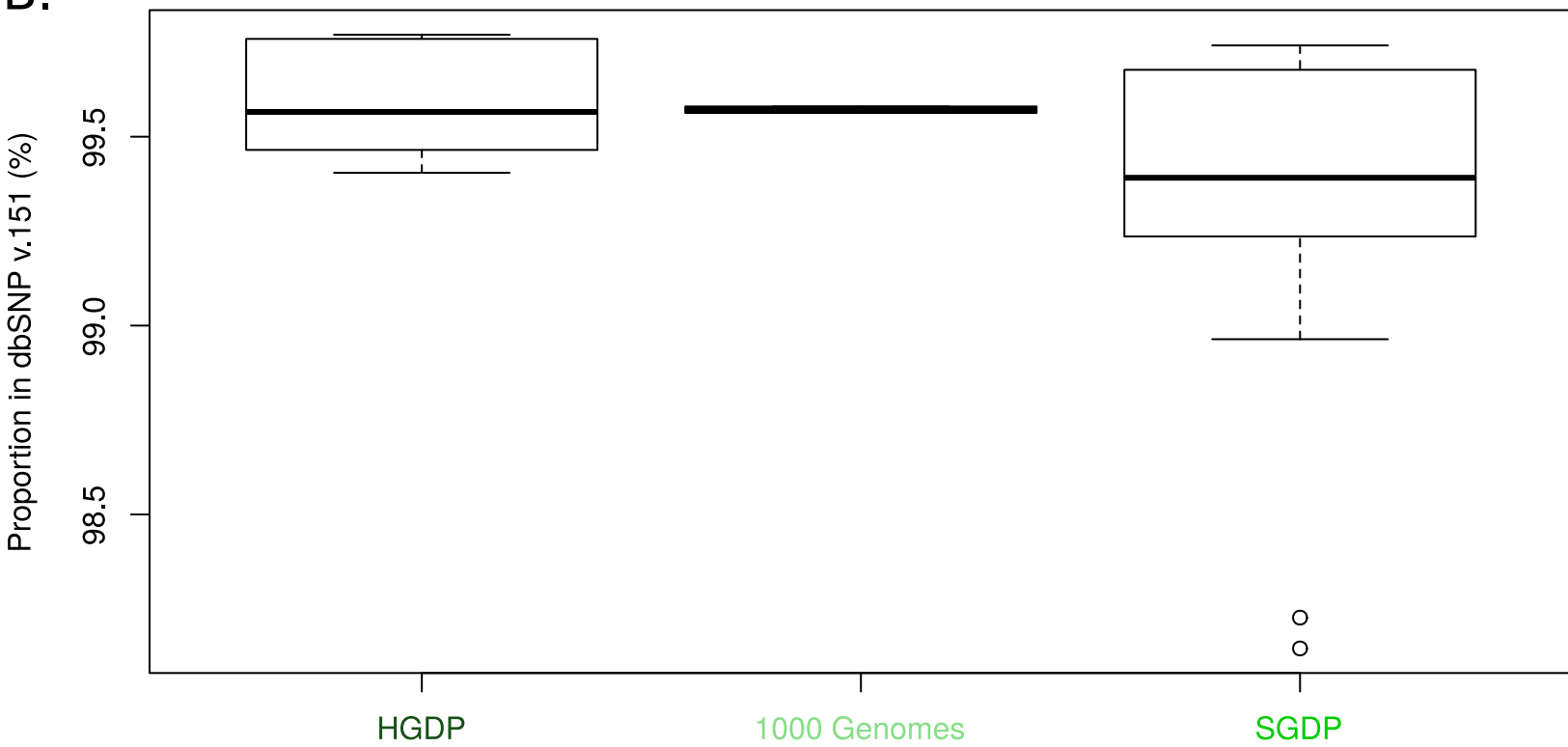

Supplement: Supplementary file 7 — Additional file 7. Box plots of the percentage of known biallelic SNPs by individual, according to ancestry or dataset. Percentage of known biallelic SNPs (relative to dbSNP v.151) in “BP2019” (before VQSR). A-Individuals are grouped by ancestry. B-Individuals are grouped by dataset [file 12859_2021_4407_MOESM7_ESM.pdf]

# Proportion of simple indels in dbSNP v.151

A.

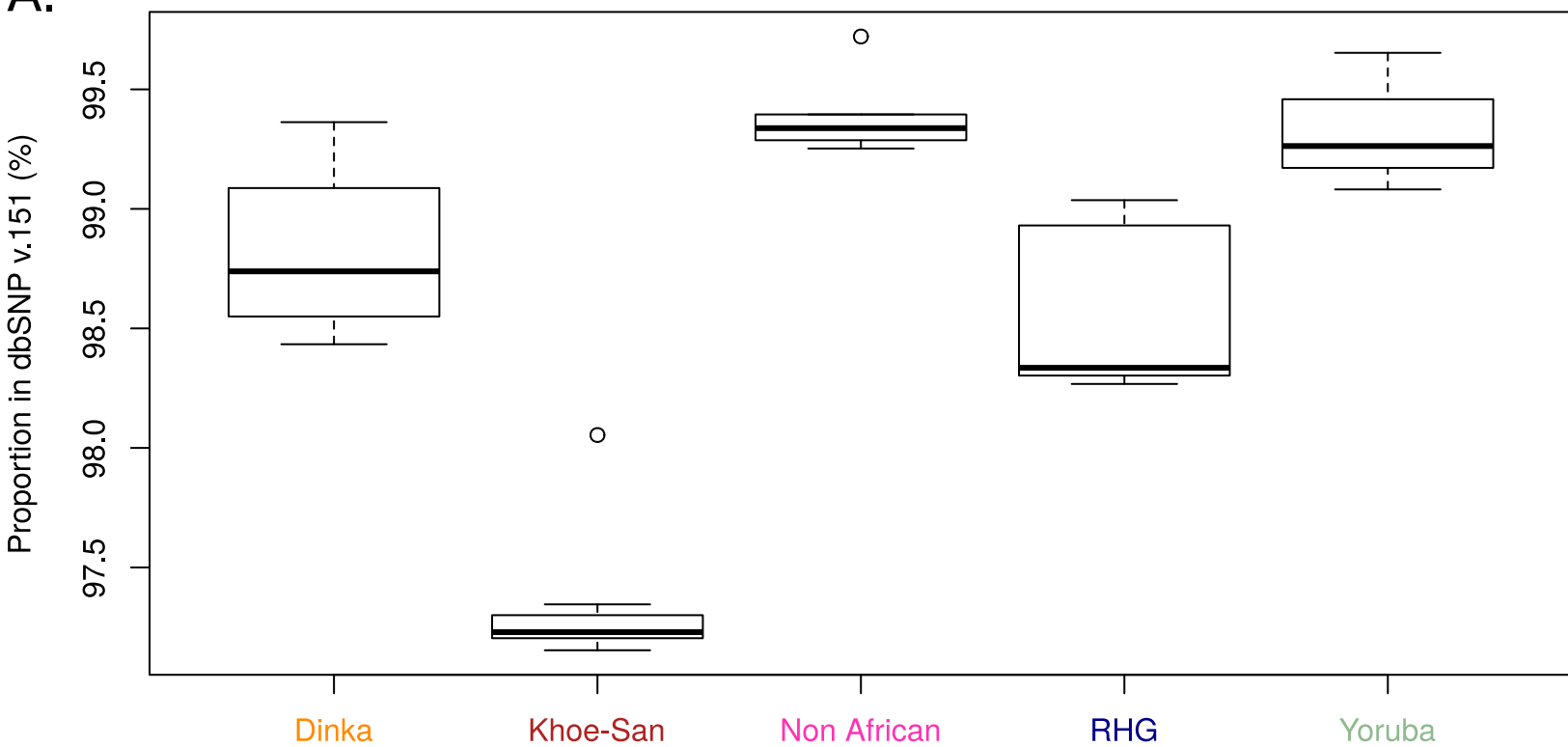

B.

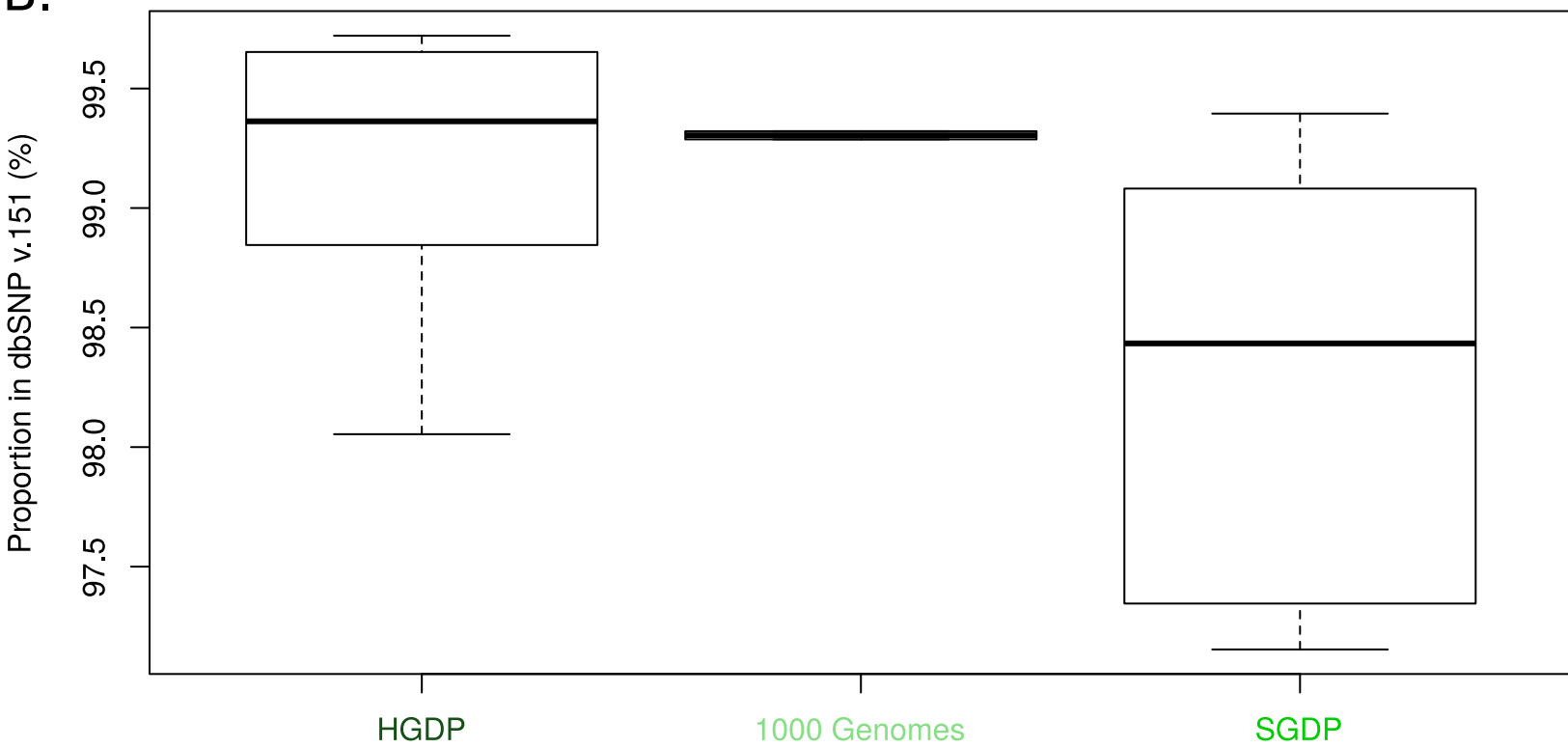

Supplement: Supplementary file 8 — Additional file 8. Box plots of the percentage of known simple indels by individual, according to ancestry or dataset. Percentage of known simple indels (relative to dbSNP v.151) in “BP2019” (before VQSR). A-Individuals are grouped by ancestry. B-Individuals are grouped by dataset [file 12859_2021_4407_MOESM8_ESM.pdf]

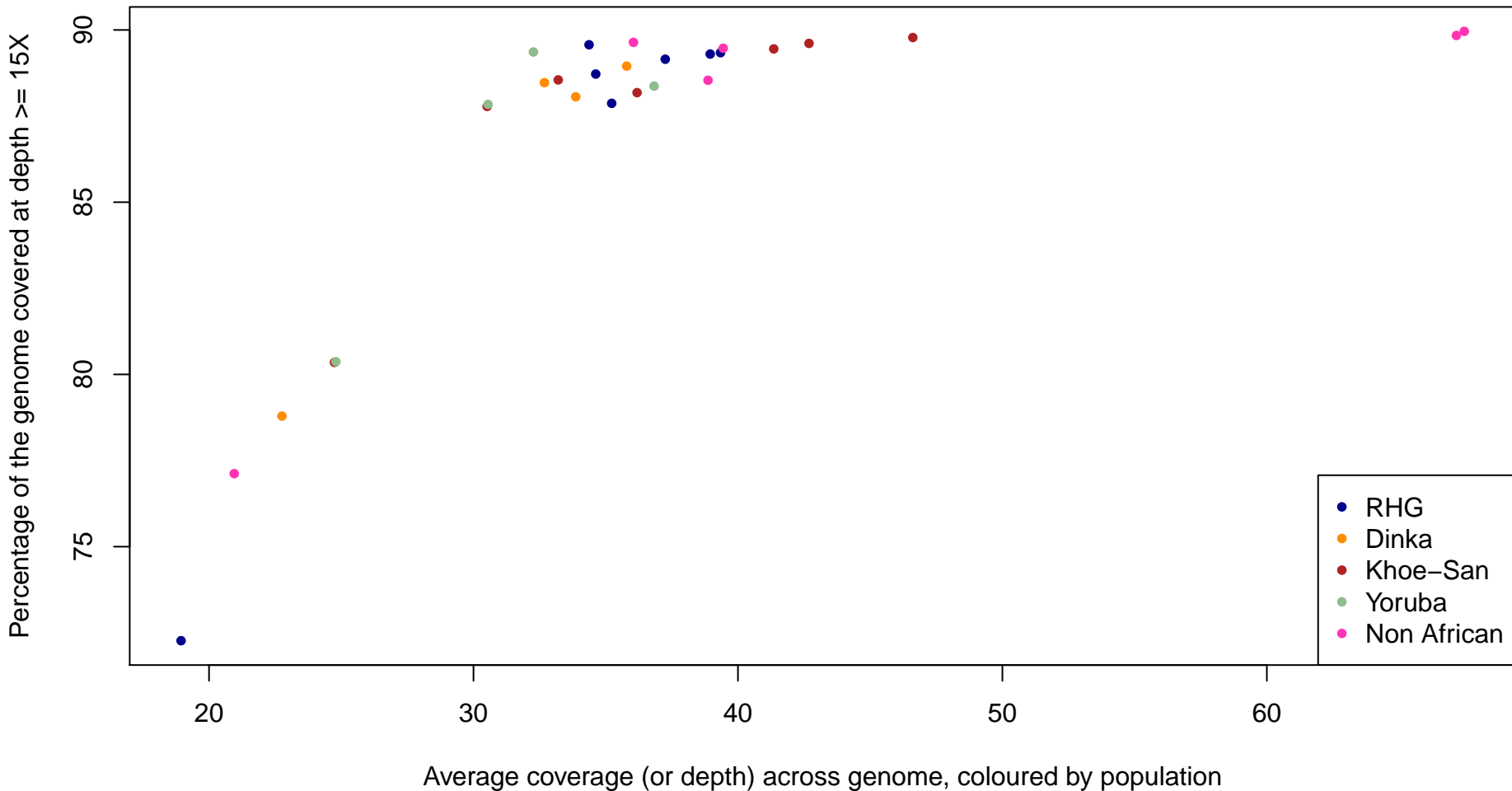

Supplement: Supplementary file 9 — Additional file 9. Genome coverage is a function of average sequencing depth. Percentage of the genome covered by at least 15X per individual in “BP2019”, against the average sequencing depth. Dots are coloured by ancestry [file 12859_2021_4407_MOESM9_ESM.pdf]
